# Virulence and genetic analysis of *Puccinia graminis tritici* in the Indian sub-continent from 2016 to 2022 and evaluation of wheat varieties for stem rust resistance

**DOI:** 10.3389/fpls.2023.1196808

**Published:** 2023-07-14

**Authors:** Pramod Prasad, Rajnikant Thakur, S. C. Bhardwaj, Siddanna Savadi, O. P. Gangwar, Charu Lata, Sneha Adhikari, Subodh Kumar, Sonu Kundu, A. S. Manjul, T. L. Prakasha, Sudhir Navathe, G. M. Hegde, B. C. Game, K. K. Mishra, Hanif Khan, Vikas Gupta, C. N. Mishra, Satish Kumar, Sudheer Kumar, Gyanendra Singh

**Affiliations:** ^1^ ICAR-Indian Institute of Wheat and Barley Research, Regional Station, Shimla, Himachal Pradesh, India; ^2^ Division of Crop Improvement, ICAR-Directorate of Cashew Research, Puttur, Karnataka, India; ^3^ ICAR-Indian Agricultural Research Institute, Indore, Regional Station, Madhya Pradesh, India; ^4^ Genetics and Plant Breeding Group, Agharkar Research Institute, Pune, India; ^5^ All India Coordinated Research Project on Wheat & Barley, University of Agricultural Sciences, Dharwad, Karnataka, India; ^6^ Mahatma Phule Krishi Vidyapeeth, Rahuri, Agricultural Research Station, Niphad, Maharashtra, India; ^7^ JNKVV, Zonal Agricultural Research Station, Powarkheda, Narmadapuram, Madhya Pradesh, India; ^8^ Crop Improvement Division, ICAR-Indian Institute of Wheat and Barley Research, Karnal, Haryana, India

**Keywords:** wheat, stem rust, *Puccinia graminis tritici*, virulence diversity, genetic diversity, rust resistance

## Abstract

Wheat stem rust, caused by *Puccinia graminis* f. sp. *tritici* (*Pgt*), has re-emerged as one of the major concerns for global wheat production since the evolution of Ug99 and other virulent pathotypes of *Pgt* from East Africa, Europe, Central Asia, and other regions. Host resistance is the most effective, economic, and eco-friendly approach for managing stem rust. Understanding the virulence nature, genetic diversity, origin, distribution, and evolutionary pattern of *Pgt* pathotypes over time and space is a prerequisite for effectively managing newly emerging *Pgt* isolates through host resistance. In the present study, we monitored the occurrence of stem rust of wheat in India and neighboring countries from 2016 to 2022, collected 620 single-pustule isolates of *Pgt* from six states of India and Nepal, analyzed them on Indian stem rust differentials, and determined their virulence phenotypes and molecular genotypes. The Ug99 type of pathotypes did not occur in India. Pathotypes 11 and 40A were most predominant during these years. Virulence phenotyping of these isolates identified 14 *Pgt* pathotypes, which were genotyped using 37 *Puccinia* spp.-specific polymorphic microsatellites, followed by additional phylogenetic analyses using DARwin. These analyses identified three major molecular groups, demonstrating fewer lineages, clonality, and long-distance migration of *Pgt* isolates in India. Fourteen of the 40 recently released Indian wheat varieties exhibited complete resistance to all 23 *Pgt* pathotypes at the seedling stage. Twelve *Sr* genes were postulated in 39 varieties based on their seedling response to *Pgt* pathotypes. The values of slow rusting parameters i.e. coefficient of infection, area under disease progress curve, and infection rates, assessed at adult plant stage at five geographically different locations during two crop seasons, indicated the slow rusting behavior of several varieties. Six *Sr* genes (*Sr2*, *Sr57*, *Sr58*, *Sr24*, *Sr31*, and *Sr38*) were identified in 24 wheat varieties using molecular markers closely linked to these genes. These findings will guide future breeding programs toward more effective management of wheat stem rust.

## Introduction

Wheat (*Triticum* spp.) with over 760 million tons of annual global production from a total area of more than 219 million hectares is one of the major food crops that provide the majority of calories worldwide (FAO, 2021). Several biotic and abiotic stresses, reducing cultivable fertile land and rapid climate change conditions, are the major limiting factors for global food production. Therefore, ensuring global food and nutrition security is frightening with a soaring world population, which is expected to reach 9 billion by 2050 (Rosegrant and Agcaoili, 2010). Among several constraints to wheat production, biotic stresses including fungal and insect damage could cause up to 21.5% yield reduction (Savary et al., 2019). Furthermore, climate change effects on temperature, relative humidity, rainfall, photoperiod, wind speed, and direction could potentially upset global wheat production directly or indirectly by maneuvering the growth, multiplication, pathogenesis, dissemination, and survival of plant pathogens (Prasad et al., 2021b).

Wheat rusts caused by *Puccinia* spp. are among the most threatening and economically important crop diseases. Stem or black rust caused by *Puccinia graminis* (Pers.) f. sp. *tritici* Eriks. & E. Henn. (*Pgt*) is one of the most detrimental diseases of wheat and has continued to threaten global wheat production since time immemorial. It can destroy all the crops if the infection happens in the early crop season and could cause 100% yield loss (Singh et al., 2011). Several stem rust epidemics were documented in the major wheat-growing regions of the world until the late 1950s (Saari and Prescott, 1985). After that era, this disease was efficiently managed through the extensive use of host resistance and eradication of alternate host, barberry (*Berberis vulgaris* L.), especially in Europe and the USA. One of the most important historical events during this period was the introduction of rye (*Secale cereale* L.) gene (1B/1R translocation), carrying *Lr26*/*Sr31*/*Yr9*/*Pm8*, into bread wheat (Mettin et al., 1973; Zeller, 1973). The 1B/1R translocation contributed 12%–20% yield jump and imparted resistance to major biotic and abiotic stresses (Cox et al., 1995). This introduction, popularly known as *Sr31* alone or with other *Sr* genes, kept the stem rust fungus under control for several decades. However, such a contentment did not last for too long and an *Sr31* virulent isolate, named TTKSK based on the North American nomenclature system and widely known as Ug99, was detected from Uganda during 1998 (Pretorius et al., 2000). The emergence of Ug99 race, virulent on at least 80% of world’s wheat varieties grown during that period, has augmented the risk of stem rust epidemics worldwide (Singh et al., 2011). The Ug99 and 15 known variants in its lineage have been reported from 14 countries in East Africa and the Middle East (https://rusttracker.cimmyt.org/?page_id=22; Singh et al., 2011; Patpour et al., 2016; Prasad et al., 2016; Nazari et al., 2021). The occurrence and movement of Ug99, its variants, and other new races caused severe localized stem rust outbreaks and epidemics in East Africa (Olivera et al., 2015), Europe (remained wheat stem rust-free since the mid- 20th century; Lewis et al., 2018), and Central Asia (Shamanin et al., 2020), indicating that stem rust is re-emerging as a challenging menace to wheat production worldwide.

Wheat stem rust is largely prevalent in nearly 7 million hectares of Central and Southern Indian states (Bhardwaj et al., 2019). India has witnessed several stem rust outbreaks in the past. Some notable epidemics were reported from Central India during 1786, 1805, 1827, 1828–1829, 1831–1832, 1879, 1894–1895, and 1907. Subsequently, a devastating stem rust outbreak was reported from the southern district of Bombay in 1946–1947 and again from Central India during 1948–1949 (Nagarajan and Joshi, 1975). Since then, stem rust was kept under control through the effective monitoring of pathogen variability and deployment of stem rust-resistant wheat cultivars and no epidemic was witnessed during last seven decades (Bhardwaj et al., 2019). Nevertheless, efforts must continue to combat possible future stem rust outbreaks due to continuous evolution in local *Pgt* races and/or exotic incursions.

Developing and deploying stem rust-resistant wheat cultivars complemented by understanding the pathogen variability and distribution is an effective, sustainable, and environmentally safe approach to manage this disease (Prasad et al., 2020a). Monitoring the pathogen variability and distribution through continuous surveillance is critical for breeding rust-resistant wheat. The *Puccinia* spp. causing wheat rusts are highly dynamic and continue to evolve, through mutation, somatic recombination, introduction, or selection, to produce new isolates periodically (Patpour et al., 2016). Such evolving nature of these pathogens renders resistant varieties susceptible in a short span. Therefore, continuous breeding for rust resistance including anticipatory breeding, search for new resistance sources, and monitoring of pathogen variability, structure, and distribution are prerequisite and continuous approaches for effective stem rust management (Abebe et al., 2012). The present study reports the following: (i) the distribution pattern of *Pgt* isolates in the Indian sub-continent from 2016 to 2022, (ii) virulence-based phenotypic and microsatellite marker-based genotypic diversity among these isolates, (iii) stem rust response of recently developed wheat varieties at seedling and adult plant stages at five different geographical locations, and (iv) the presence of *Sr* genes in these varieties postulated through gene matching technique and genetic analysis using closely linked molecular markers to these genes.

## Materials and methods

### Pathogenic and genetic diversity of *Puccinia graminis tritici*

#### Sampling and isolation of single-pustule fungal isolates

Wheat stem rust surveys were conducted from 2016 to 2022 in geographically different locations of Gujarat, Karnataka, Madhya Pradesh, Maharashtra, Tamil Nadu, and Uttarakhand states in India and Nepal. A total of 620 individual uredinial isolates of *Pgt* were collected from commercial wheat fields, trap plots, and experimental plots across these locations. All isolates were inoculated and multiplied on a stem rust-susceptible wheat cultivar, Agra Local, as described previously (Prasad et al., 2021a). To ensure the purity of individual isolates and to minimize the chances of heterogeneity within individual isolates, a well-demarcated pustule from inoculated Agra Local was excised aseptically and uredospores from it were used to inoculate new plants using a lancet needle. The inoculated plants, after 48 h of incubation in high-humidity chambers, were transferred to separate glass house chambers maintained at 25 ± 2°C. Uredospores from each isolate were collected 15 days post-inoculation (dpi) for further studies.

#### Identification of virulence phenotypes and pathotype designation

To determine the virulence phenotypes and pathotype designation of *Pgt* isolates, Indian stem rust differentials (Bahadur et al., 1985; Nayar et al., 1997; Prasad et al., 2018) were used. Indian stem rust differentials comprised 24 wheat genotypes including a universal susceptible, a line resistant to all pathotypes that serves as a control, currently cultivated wheat lines, individual *Sr* genes, and lines from the international stem rust differential set. Three to five seedlings of these differentials were raised to the single-leaf stage and spray inoculated with a uredospore suspension containing 5 mg uredospores/ml of non-phytotoxic isoparaffinic mineral oil Soltrol 170 (Chevron Phillips Chemical Company, USA). The inoculated seedlings were sprayed with a fine mist of water, and incubated in high-humidity chambers for 24 h. Subsequently, inoculated plants were transferred to the glasshouse chambers as described previously. The virulence phenotypes of each isolate on stem rust differentials were determined at 15 dpi using a 0–4 infection rating scale (Stakman et al., 1962) with some modifications (Nayar et al., 1997), where scores 0–2 and 3–4 were considered as avirulent and virulent reactions, respectively. Repeated virulence phenotyping of each *Pgt* isolate was done to confirm their identity. Pathotypes were designated according to their avirulence/virulence profiles on Indian stem rust differentials (Nagarajan et al., 1983). All 14 pathotypes identified in 620 *Pgt* isolates detected during the study were mass multiplied on Agra Local in separate chambers. The harvested uredospores were shade dried and stored at −20°C for genetic analysis.

#### SSR genotypes and phylogenetic analysis

Genetic analysis of 14 *Pgt* pathotypes (11, 11A, 15-1, 21, 21-1, 21A-2, 34-1, 40A, 40-2, 40-3, 42, 117, 117-6, and 122), identified during 2016–2022, was performed using *Puccinia* spp.-specific microsatellite/simple sequence repeat (SSR) markers. The genomic DNA was isolated from dried uredospores (~50 mg) of each pathotype using the cetyltrimethylammonium bromide (CTAB) method (Kiran et al., 2017). The quality and quantity of genomic DNA were determined using NanoDrop 2000^®^ UV-Vis Spectrophotometer (Thermo Fisher Scientific, Waltham, Massachusetts, USA). The quality of genomic DNA was also assessed through agarose gel electrophoresis (1% agarose). A set of 100 *Puccinia* spp.-specific SSR markers designed previously (Karaoglu et al., 2013; Prasad et al., 2017; Prasad et al., 2018; Savadi et al., 2020), synthesized in Agile Life Science Technologies (New Delhi, India), were used for genotyping 14 *Pgt* pathotypes. Thirty-seven markers (**Supplementary Table 1**), identified as polymorphic in our earlier study (Prasad et al., 2022), were selected to characterize *Pgt* pathotypes. Each 20 μl of the polymerase chain reaction (PCR) reaction mixture contained 25 ng of template DNA, 800 μM of dNTPs, 1X PCR buffer (10 mM Tris, pH 9.0, and 50 mM KCl), 1.5 mM MgCl_2_, 0.5 U Taq polymerase (HiMedia Laboratory Pvt. Ltd., Mumbai, Maharashtra, India), and 10 pmol of both forward and reverse primers. The reaction programs were set at 94°C for 2 min, followed by 35 cycles of 30 s at 94°C, 30 s at 48°C, and 1 min at 72°C, with a final extension at 72°C for 10 min in a thermal cycler (Applied Biosystems Veriti™, California, USA). After the completion of amplification, the amplified DNA was analyzed on 3% Super MT4 Agarose gel (Life Technologies India Pvt. Ltd., New Delhi, India) in 1X TBE buffer at 65–70 V for 2–3 h. DNA fragments were visualized under UV light and photographed using the gel documentation system (Bio-Rad Laboratories Inc., Hercules, California, USA).

### Assessment of Indian wheat varieties for stem rust resistance

#### Plant and pathogen material

A set of 40 wheat varieties (Bread and Durum) recently released for commercial cultivation in India was evaluated. The central varietal release committee identified and notified these varieties based on several parameters, including yield advantage, disease resistance, and quality. The parentage, year of release, and characteristic features of these varieties are presented in **Table 1**. Twenty-three pathotypes (**Supplementary Table 2**) of *Pgt* with different avirulence/virulence formula were used to assess the seedling stage stem rust resistance and postulate stem rust resistance genes among selected 40 wheat varieties (**Table 1**). These pathotypes are being maintained in the national cereal rusts pathogen repository at the Indian Council of Agricultural Research–Indian Institute of Wheat and Barley Research (ICAR-IIWBR), Regional Station, Shimla, India.

**Table 1:** List of the tested Indian wheat varieties and their pedigrees, year of release, and characteristic features.

S. No.	Variety/Line	Pedigree	Year of release	Characteristic features
1	CG1029	HW2004/PHS725	2021	Good Chapatti quality and tolerance to heat stress
2	CG1036	HW2004/PHS832	2022	Yield advantage and superior grain quality
3	DBW252	PFAU/MILAN/5/CHEN/AE.SQ(TAUS)//BCN/3/VEE#7/BOW/4/PASTOR	2020	Resistance to wheat blast, leaf rust, and karnal bunt
4	DDW47 (d)*	PBW34/RAJ1555//PDW314	2020	Resistance to stem and leaf rusts with high pasta score
5	DDW48 (d)	HI8498/PDW233//PDW291	2021	High pasta score
6	HD2733	ATTILA/3/TUI/CARC//CHEN/CHTO/4/ATTILA	2001	Resistance to leaf rust and tolerance to leaf blight
7	HD2967	ALD/COC//URES/HD216 0M/HD2278	2011	Wider adaptability and resistance to stripe (yellow) and leaf rusts
8	HD3043	PJN/BOW//OPATA*2/3/CROC_1/*Ae. squarrosa* (224)//OPATA	2012	Adult plant resistance to stripe and leaf rusts
9	HD3090	SFW/VAISHALI//UP2425	2014	Resistance to leaf and stem rust
10	HD3118	ATTILA*2/PBW65//WBLL1*2/TUKURU	2015	High degree of resistance to stripe and leaf rust
11	HD3171	PBW 343/HD 2879	2017	High degree of resistance to stripe, leaf, and stem rusts, higher iron content (47.1 ppm)
12	HD3249	PBW343*2/KUKUNA//SRTU/3/PBW343*2/KHVAKI	2020	High degree of resistance to leaf rust and wheat blast
13	HD3293	HD2967/DBW46	2021	Resistance to wheat blast
14	HI1621	W15.92/4/PASTOR//HXL7573/2*BAU/3/WBLL1	2020	High degree of resistance to leaf rust and stripe rust
15	HI1628	FRET2*2/4/SNI/TRAP#1/3/KAUZ*2/TRAP//KAUZ/5/PFAU/ WEAVER//BRAMBLING	2020	High degree of resistance to leaf and stripe rusts
16	HI1633	GW322/PBW498	2021	Grain protein (12.4%), iron = 41.6 ppm, and zinc = 41.1ppm
17	HI1634	GW322/PBW498	2021	Good Chapatti quality, resistant to leaf and stem rusts
18	HI1650	Giant3/HI1395	2022	Yield gains and superior grain quality
19	HI1653	NADI/COPIO//NADI	2022	Resistance to leaf and stem rusts
20	HI1654	SOKOLL/3/PASTOR//HXL7573/2*BAU/4/PANDION//FILIN/2* PASTOR/3/BERKUT	2022	Resistance to leaf and stem rusts
21	HI1655	MACS2496/HI1531	2022	Resistance to leaf and stem rusts
22	HI8498 (d)	CR “S’-GS’S’/A - 9-30-1//RAJ911	1999	Resistance to leaf and stem rust, suitable for suji and dalia
23	HI8826 (d)	HI8713/HI8663	2022	Resistance to leaf and stem rusts, yield superiority
24	HI8830 (d)	HI8713/HI8663	2022	Yield advantage and resistance to stem and leaf rusts
25	HPW349	OASIS/SKAUZ//4*BCN/3/PASTOR/4/KAUZ*2/YACO//KAUZ	2013	Resistance to stripe rust, leaf rust, and good for chapatti quality
26	HS507	KAUZ/MYNA/VUL/BUC/FLK/4/MILAN	2011	Resistance to leaf rust, stripe rust, leaf blight, and karnal bunt
27	HS562	OASIS/SKAUZ//4*BCN/3/2*PASTOR	2016	Field resistance to leaf and stripe rusts
28	KRL19	PBW255/KRL 1-4	2000	Suitable for water logging area and resistance to stripe and leaf rusts
29	KRL210	PBW65/2*PASTOR	2012	Resistance to stripe rust and karnal bunt
30	MACS4100 (d)	CBC509CHILE/6/ECO/CMH76A.722//BIT/3/ALTAR84/4/AJAIA_2/5/ KJOVE_1/7/AJAIA_12/F3LOCAL(SEL.ETHIO.135.85)//PLATA_13/8 /SOOTY_9/RASCON_37//WODUCK/CHAM_3	2022	Resistance to leaf and stem rusts, yield superiority
31	MACS6768	MACS6221*2/Raj4037	2022	Yield gains and superior grain quality
32	MP3288	DOVE/BUC/DL 788-2	2011	Tolerance to major diseases
33	MP3336	HD 2402/GW 173	2013	Resistance to leaf and stem rusts
34	NIAW3170	SKOLL/ROLF07	2021	Soft grains, biscuit spread factor
35	NIDW1149 (d)	NIDW295/NIDW15	2021	Resistance to leaf and stripe rusts
36	PBW771	PBW550//YR15/6*AVOCET/3/2*PBW550	2020	High resistance to leaf and stripe rust
37	RAJ4083	PBW 343/UP2442//WR 258/UP2425	2007	Resistance to leaf and stem rusts
38	VL2041	NESSER/SAULSKU32/MACS6240//HS507	2022	Superior grain quality and suitability for biscuit making
39	VL907	DYBR 1982- 83/842 ABVD50/VW9365//PBW 343	2010	Resistance to stripe rust and leaf rust, and possess high iron content
40	WH1124	MUNIA/CHTO/AMSEL	2014	Resistance to stripe rust and leaf rust, and tolerance to terminal heat
41	Agra Local	–	–	Susceptible check

*(d) denotes durum wheat.

#### Seedling response and postulation of stem rust resistance genes

Four to six seeds of each variety and a standard set of 24 stem rust differentials were sown in 10-cm pots filled with a mixture of fine loam and farmyard manure (3:1) in an environmentally controlled greenhouse at ICAR-IIWBR, Regional Station, Shimla. Stem rust differentials were included to determine the purity of all pathotypes. Each pot had four varieties in a clockwise circle. After sowing, pots were kept in isolated rust-free greenhouses at 20°C. All wheat varieties and 24 stem rust differentials were screened against 23 pathotypes of *Pgt* (**Supplementary Table 2**). The uredospore suspension (10 mg uredospores/5 ml of Soltrol) was used to inoculate 1-week-old seedlings using a glass atomizer. The inoculated seedlings were then transferred to dew chambers at 25 ± 2°C and 90%–100% relative humidity (RH) and incubated for 48 h. Subsequently, the inoculated plants were transferred to temperature and RH-controlled separate greenhouse benches maintained at 25 ± 2°C and 60%–70% RH. Seedling infection types (ITs) were recorded at 14 dpi using a 0–4 scale (Stakman et al., 1962) with slight modifications as proposed by Luig (1983). IT 0 (immune or fleck), 1 (small uredia with necrosis), and 2 (small to medium uredia with chlorosis or necrosis) indicate incompatibility, whereas IT 3 (medium-size uredia with/without chlorosis) and 4 (large uredia without chlorosis or necrosis) specify compatible interaction between a specific host and pathotype. This experiment was repeated to validate the response of these varieties against all *Pgt* pathotypes. The seedling response (ITs) of all 40 varieties to 23 pathotypes of *Pgt* was utilized to postulate *Sr* genes. The gene postulation was done using the gene matching technique by comparing IT arrays of all the pathotypes on test variety with those of controls with known resistance genes (Browder, 1973; Nayar et al., 1997). In case of *Sr31*, linkage with *Lr26*/*Yr9* was used to infer resistance. Furthermore, micro flecking, the morphological marker linked to *Sr2*, was also utilized for postulating *Sr2* gene (Khan et al., 2017).

#### Adult plant response

Adult plant stem rust response (APR) of 40 test wheat varieties and a susceptible check (Agra Local) was investigated during two crop seasons 2020–2021 and 2021–2022 at the following five geographically different stem rust hot spot locations (experimental farm): (i) ICAR-Indian Agriculture Research Institute, Regional Station, Indore, Madhya Pradesh (GPS coordinates: 22°42′31.7′′N, 75°53′29.0′′E); (ii) Jawaharlal Nehru Krishi Vishwavidyalaya, Zonal Agricultural Research Station, Powarkheda, Madhya Pradesh (GPS coordinates: 22°42′03.8′′N, 77°44′49.8′′E); (iii) Agharkar Research Institute, Hol farm, Baramati, Maharashtra (GPS coordinates: 18°07′07.8′′N, 74°18′26.8′′E); (iv) Mahatma Phule Krishi Vidyapeeth, Agricultural Research Station, Niphad, Maharashtra (GPS coordinates: 20°06′20.7′′N, 74°04′26.7′′E); and (v) University of Agricultural Sciences, Dharwad, Karnataka (GPS coordinates: 15°29′29.6′′, 74°58′34.0′′E). The well-prepared and drained fields were used and recommended doses of fertilizers and other agronomic practices for respective wheat production areas were followed. Approximately 30 g of seeds/row of each test entry was sown during the regular sowing period in three rows of 3 m with 20-cm row spacing. The experiments were laid out in randomized block design with three replicates during both the crop seasons at all five locations. A double row of a spreader mixture of two universal stem rust-susceptible wheat cultivars Agra Local and Lal Bahadur was planted perpendicular to all three replicate blocks and a single row after every five test entries for each block. The uredospores suspension of most virulent and predominant *Pgt* pathotypes 11, 21A-2, 40A, 117-6, and 122, collected from ICAR-IIWBR, Shimla, was prepared in distilled water mixed with 0.05% Tween 20 for inoculation. Artificial stem rust epiphytotic was initiated by spray inoculation of disease spreaders twice at three and four leaf stages, respectively, with freshly prepared uredospores suspension. A fine distilled water spray was frequently done on and around inoculated spreader plants until 48 hours to get desired RH for optimum spore germination and infection.

The adult plant stem rust response of all test entries was scored as infection type and disease severity when the spreaders had 50% disease severity. Disease response and severity were scored three times at weekly intervals. The response of the test entries was expressed in six infection types (Johnston and Browder, 1966) as follows: 0 (No infection, score 0.0), R (Resistant, necrotic areas with or without minute uredia, 0.2), MR (Moderately resistant, small uredia surrounded by necrotic areas, 0.4), X (Intermediate, variable size uredia, some with necrosis or chlorosis, 0.6), MS (Moderately susceptible, medium uredia with no chlorosis, 0.8), and S (Susceptible, large uredia, no necrosis or chlorosis, 1.0). A modified Cobb scale was used to determine the percentage coverage of stem/leaves with stem rust pustules (Peterson et al., 1948).

### Identification of *Sr* genes using molecular markers

Genomic DNA was extracted from fresh leaves of 40 wheat varieties and positive and negative controls for all screened *Sr* genes using the CTAB method (Bansal et al., 2010). The quality and quantity of genomic DNA were determined using NanoDrop 2000^®^ UV-Vis Spectrophotometer (Thermo Fisher Scientific, Waltham, Massachusetts, USA). The presence of four slow rusting/adult plant response (APR) (*Sr2*/*Yr30*, *Sr57/Lr34/Yr18, Sr58/Lr46/Yr29*, and *Sr55/Lr67/Yr46*) and three all stage (ASR) *Sr* genes (*Sr24/Lr24*, *Sr31/Lr26/Yr9*, and *Sr38/Lr37/Yr17*) was determined in 40 wheat varieties using closely linked molecular markers to these genes (**Table 2**). The PCR was performed in 20 μl of reaction mixture containing 25 ng of template DNA, 200 μM of dNTPs, 1X PCR buffer (10 mM Tris, pH 9.0, and 50 mM KCl), 1.5 mM MgCl_2_, 2.5 U Taq polymerase (Hi Media Lab, Mumbai, India), and 10 pmol of both forward and reverse primers. The PCR programs were set at 94°C for 5 min (initial denaturation), followed by 35 cycles of 30 s at 94°C, 30 s at marker specific annealing temperature (**Table 2**), and 1 min at 72°C, with a final extension at 72°C for 7 min in a thermal cycler (Applied Biosystems Veriti™, California, USA). A touchdown PCR profile (annealing temperature reduced by 1°C/cycle for first 5 cycles) was used for Xgwm533 marker (Spielmeyer et al., 2003). The PCR products were resolved on 3% high-resolution Agarose (Hi Media Lab, Mumbai, India) gel using 1× TAE buffer and pre-stained with ethidium bromide. Gel imaging was done in gel documentation system (Bio-Rad Laboratories Inc., Hercules, California, USA). The allele scoring was performed using Gene Mapper v 4.0 software (Applied Biosystems, California, USA). Separate negative and positive controls were included for each marker analysis and observations were repeated to confirm the results.

**Table 2:** Detail of DNA markers used to identify the presence of *Sr* genes in wheat varieties.

Gene (s)	Linked molecular marker	Sequence (5′–3′)	AT* (°C)	Expected fragment size (bp) for positive	Reference
*Sr2*/*Yr30*	Xgwm533	F AAGGCGAATCAAACGGAATA R GTTGCTTTAGGGGAAAAGCC	60 TD**	120	Spielmeyer et al., 2003
*Sr55/Lr67/Yr46*	CFD71	F: CAATAAGTAGGCCGGGACAA R: TGTGCCAGTTGAGTTTGCTC	52	148	Hiebert et al., 2010
*Sr57/Lr34/Yr18*	*csLV34*	F: GTTGGTTAAGACTGGTGATGG R: TGCTTGCTATTGCTGAATAGT	55	150	Lagudah et al., 2006
*Sr58/Lr46/Yr29*	WMC44	F: GGTCTTCTGGGCTTTGATCCTG R: TGTTGCTAGGGACCCGTAGTGG	58	242	Rosewarne et al., 2006
*Sr24/Lr24*	Sr24#50	F: CCCAGCATCGGTGAAAGAA R: ATGCGGAGCCTTCACATTTT	63	200	Spielmeyer et al., 2003
*Sr31*/*Lr26*/*Yr9*	iag95	F: CTCTGTGGATAGTTACTTGATCGA R: CCTAGAACATGCATGGCTGTTACA	55	1,100	Mago et al., 2005
*Sr38*/*Lr37*/*Yr17*	VENTRIUP-LN2	F: AGGGGCTACTGACCAAGGCT R: TGCAGCTACAGCAGTATGTACACAAAA	58	259	Helguera et al., 2003

*AT, annealing temperature; ** touchdown profile for annealing.

### Data analysis

Based on SSR allele sizes, a multilocus allelic data matrix for each pathotype–primer combination was generated as binary data for the alleles’ presence (1) or absence (0). Polymorphic information content (PIC) value, distinguishing two locus or loci, was assessed following Anderson et al. (1993):


$$PIC_{j}=1-\sum_{f=1}^{n}{\left(P_{ij}\right)}^{2}$$

where *n* and $P_{ij}$ are the numbers of alleles and frequency of the *j*th allele for marker *f*, respectively.

Based on the SSR genotype data, the genetic relationship among the *Pgt* pathotypes was determined using an unrooted dendrogram generated by unweighted neighbor-joining (NJ) cluster analysis in DARwin 6.0.14 (Perrier and Jacquemoud-collet, 2006). A total of 3,000 bootstraps were used to analyze the data set in DARwin 6.0.14 with no missing data option.

Stem rust resistance behavior of all test entries was assessed through the average coefficient of infection (ACI), area under disease progress curve (AUDPC), and infection rate (*r*-value). The average coefficient of infection (ACI) was calculated following Saari and Wilcoxson (1974). AUDPC was calculated by using the formula given by Wilcoxson et al. (1975).


$$AUDPC=\left[0.5\left(X_{i}+\;X_{i+1}\right)\right]\left[t_{i+1}-t_{i}\right]$$

where *X_i_* = the average coefficient of infection of the *i*th record, *X_i_*_+1_ = the average coefficient of infection of the i+1^th^ record, *t _i_*_+1 −_ *t_i_* = number of days between the *i*th record and *i*+1^th^ record, and *n* = number of observations. The ACI and AUDPC scores were used to perform the Wilcoxon signed-rank test to rank wheat varieties based on their APR to stem rust (Xia, 2020).

The infection rate was estimated using the following formula proposed by Van der Plank (1963).


$$r-\text{Value}=\frac{1}{t_{2}-t_{1}}\left({\text{log}}_{\text{e}}\frac{X_{2}}{1-X_{2}}-{\text{log}}_{\text{e}}\frac{X_{1}}{1-X_{1}}\right)$$

where *X*_1_ = the proportion of the infected leaf area (disease severity) at date *t*_1_, *X*_2_ = the proportion of the infected leaf area (disease severity) at date *t*_2_, and *t*_2_ − *t*_1_ = the interval in days between these dates.

## Results

### Occurrence of wheat stem rust from 2016 to 2022

**Figure 1:**
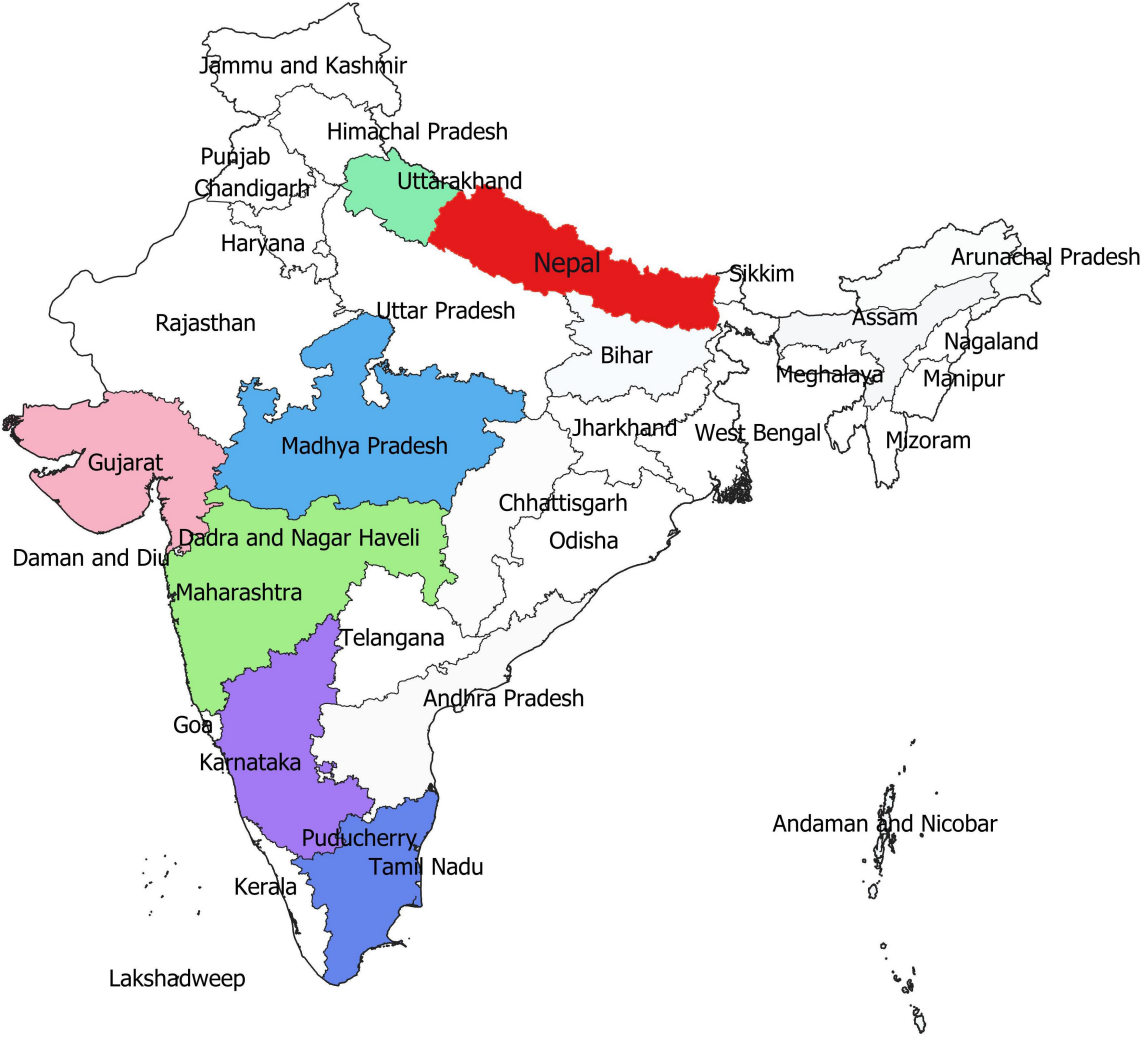
The distribution of wheat stem rust (highlighted in colors) in different wheat-growing areas of India and Nepal during 2016–2022.

**Figure 2:**
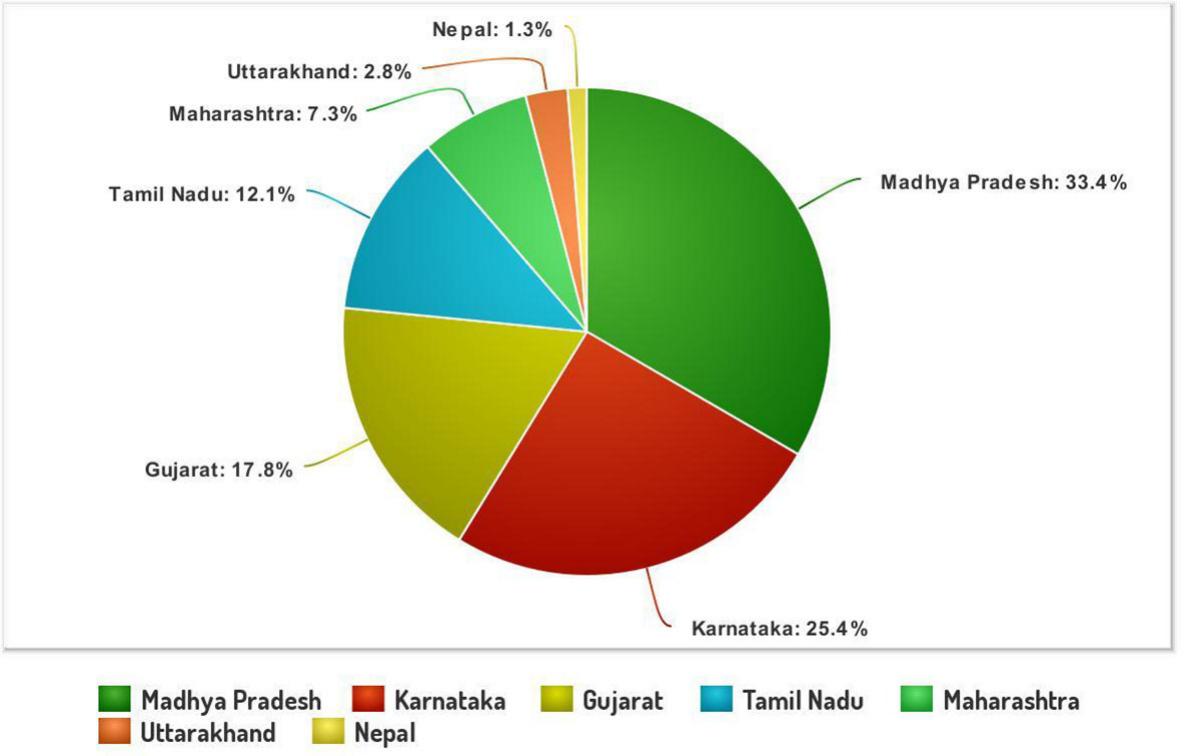
The stem rust samples (%) collected and analyzed from different Indian states and Nepal during 2016–2022.

**Figure 3:**
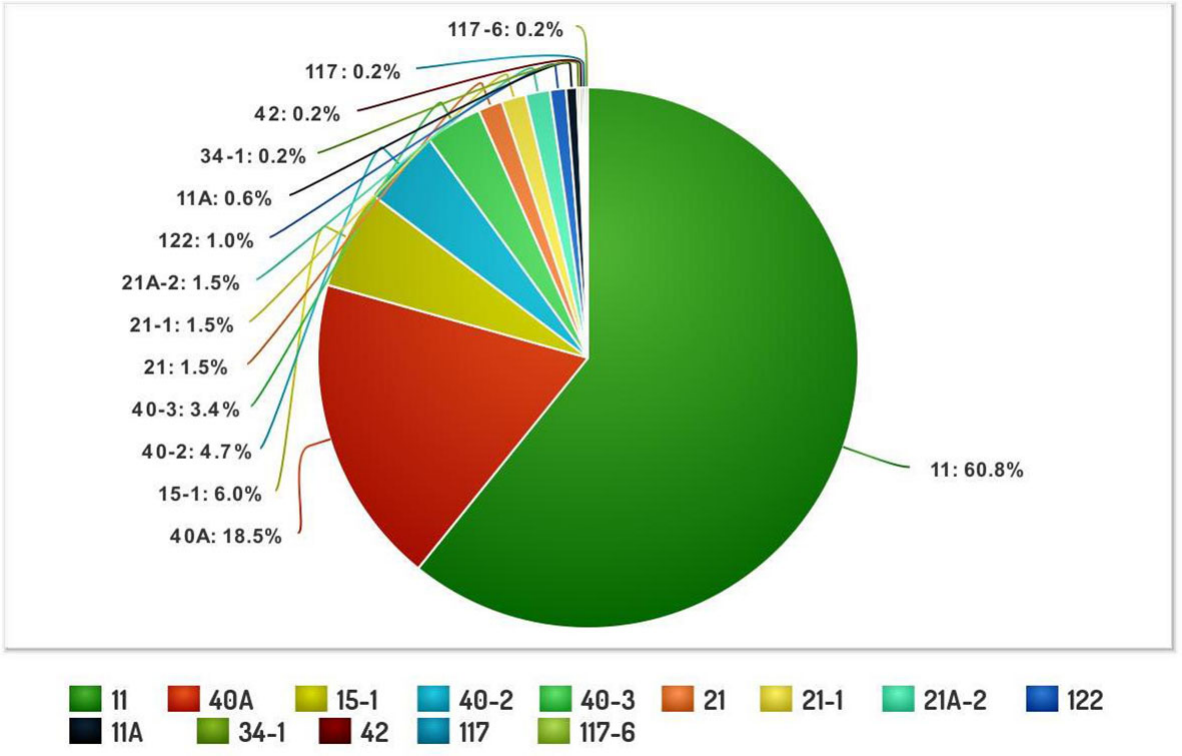
The composite frequency (%) of *Puccinia graminis* f. sp. *tritici* pathotypes identified from major wheat-growing regions of India and Nepal during 2016–2022.

The systematic monitoring conducted during main and off-seasons in major stem rust-prone areas indicated that the distribution of wheat stem rust was not uniform. The natural infection of stem rust was observed only in six Indian states (Gujarat, Madhya Pradesh, Maharashtra, Karnataka, Tamil Nadu, and Uttarakhand) and Nepal (**Figure 1**). The occurrence of stem rust was usually observed towards the crop maturity, and therefore, no stem rust epidemic was reported during this period from India or neighboring countries. The stem rust severity was low (up to 30 MS to S) on advanced wheat varieties, whereas off-types, old wheat genotypes, and other susceptible varieties suffered up to 80 S severity. Six-hundred twenty samples of wheat stem rust were collected during this period and analyzed on Indian stem rust differentials. The incidence of stem rust, reported only from Gujarat, Madhya Pradesh, Maharashtra, and Tamil Nadu, was very low during 2021–2022. The overall prevalence of wheat stem rust was maximum in Madhya Pradesh, followed by Karnataka, Gujarat, and Tamil Nadu as 33.4%, 25.4%, 17.8%, and 12.1% stem rust-infected samples, respectively, were collected from these states (**Figure 2**). Ug99 types of pathotypes were not observed in any sample. The pathotype analysis resulted in the identification of 14 *Pgt* pathotypes (11, 11A, 15-1, 21, 21-1, 21A-2, 34-1, 40A, 40-2, 40-3, 42, 117, 117-6, and 122) in these samples. Pathotypes 11 and 40A were the most predominant and were reported in 60.8% and 18.5% of samples (**Figure 3**), respectively. These two pathotypes were observed during all six crop seasons. Other pathotypes were missing at least in one crop season. The frequency (%) of pathotype 11, ranging from 43.05 (2016–2017) to 88.18 (2019–2020), was maximum during all six crop seasons. It was observed in all the states where stem rust appeared, whereas pathotype 40A was present in Madhya Pradesh, Karnataka, and Tamil Nadu only. The frequency (%) of pathotype 40A ranged between 4.72 (2019–2020) and 47.22 (2016–2017). Pathotypes 34-1, 42, 117, and 117-6 were observed only in one sample during 2021–2022, 2019–2020, 2017–2018, and 2017–2018, respectively (**Table 3**). Pathotypes 15-1, 40-2, and 40-3 were identified in 6%, 4.7%, and 3.4% of samples, respectively.

**Table 3:** Year-wise distribution and frequency of *Puccinia graminis* f. sp. *tritici* pathotypes identified from major wheat-growing regions of India and Nepal during 2016–2022.

Pathotypes	Year	Number of pathotypes								Frequency (%)
		Gujarat	Karnataka	Madhya Pradesh	Maharashtra	Tamil Nadu	Uttarakhand	Nepal	Total	
11 (79G31*, RRTSF**)	2015–2016	17	0	06	0	01	04	0	28	51.85
	2016–2017	22	0	0	06	03	0	0	31	43.05
	2017–2018	08	26	07	07	0	0	0	48	60.00
	2018–2019	33	30	02	0	01	0	01	67	50.00
	2019–2020	14	25	56	16	0	0	01	112	88.18
	2020–2021	01	01	45	01	0	0	0	48	52.74
	2021–2022	18	0	24	01	0	0	0	43	69.35
11A (203G15, RHTSF)	2017–2018	02	0	0	01	0	0	0	3	3.75
	2019–2020	0	0	0	01	0	0	0	1	0.78
15-1 (123G15, TKTSF)	2018–2019	0	30	0	0	0	0	0	30	22.38
	2019–2020	0	0	0	01	0	0	0	1	0.78
	2020–2021	0	0	0	0	06	0	0	6	6.59
21 (9G5, CHMQC)	2017–2018	0	0	0	0	0	0	01	1	1.25
	2018–2019	0	01	0	0	0	0	0	1	0.74
	2020–2021	0	03	0	0	0	0	0	3	3.29
	2021–2022	01	0	03	0	0	0	0	4	6.45
21-1 (24G5, CKMSC)	2015–2016	0	0	0	0	0	04	0	4	7.40
	2016–2017	0	01	0	0	01	0	0	2	2.77
	2017–2018	0	0	0	0	0	0	02	2	2.5
	2020–2021	0	01	0	0	0	0	0	1	1.09
21A-2 (75G5, CCTJC)	2015–2016	0	0	0	0	0	06	0	6	11.11
	2018–2019	0	01	0	0	0	0	0	1	0.74
	2020–2021	0	0	0	0	02	0	0	2	2.19
34-1 (10G13, MCGGP)	2021–2022	0	0	01	0	0	0	0	1	1.61
40A (62G29, PTHSC)	2015–2016	0	0	0	0	09	0	0	9	16.66
	2016–2017	0	0	0	0	34	0	0	34	47.22
	2017–2018	0	03	02	0	19	0	0	24	30
	2018–2019	0	02	0	0	19	0	0	21	15.67
	2019–2020	0	0	0	0	06	0	0	6	4.72
	2020–2021	0	0	0	0	14	0	0	14	15.38
	2021–2022	0	0	0	0	07	0	0	7	11.29
40-2 (58G13-3, PKRSC)	2015–2016	0	0	01	04	0	0	0	5	9.25
	2018–2019	0	08	0	0	0	0	0	8	5.97
	2019–2020	0	01	03	0	01	0	0	5	3.93
	2020–2021	0	0	0	0	04	0	0	4	4.39
	2021–2022	0	0	0	0	07	0	0	7	11.29
40-3 (127G29, PTKSF)	2015–2016	0	0	0	0	01	0	0	1	1.85
	2016–2017	0	0	0	0	02	0	0	2	2.77
	2018–2019	0	02	0	0	03	0	0	5	3.73
	2020–2021	0	0	0	0	13	0	0	13	14.28
42 (19G35, HKGGC)	2019–2020	0	01	0	0	0	0	0	1	0.78
117 (37G3, KRCSC)	2017–2018	01	0	0	0	0	0	0	1	1.25
117-6 (37G19, KRCSC)	2017–2018	0	01	0	0	0	0	0	1	1.25
122 (7G11, RRJQC)	2015–2016	0	0	0	0	01	0	0	1	1.85
	2016–2017	0	0	02	0	01	0	0	3	4.16
	2018–2019	0	01	0	0	0	0	0	1	0.74
	2019–2020	0	0	0	0	0	0	01	1	0.78

*Indian binomial pathotype notation (Bahadur et al., 1985).

** North American race notation (Jin et al., 2008).

### SSR genotypes and phylogenetic analysis

**Figure 4:**
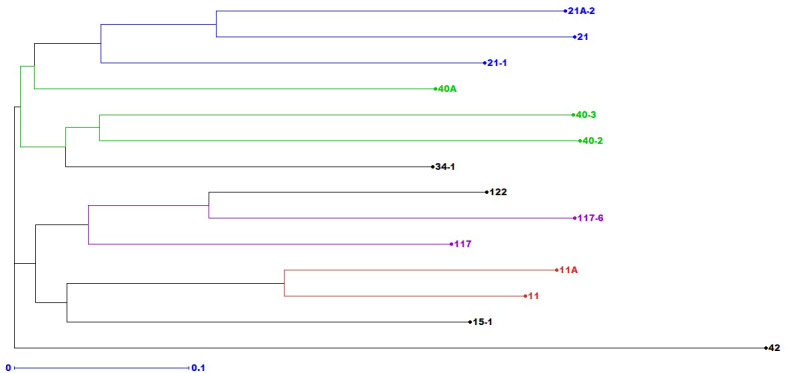
The simple sequence repeats-based unrooted neighbor-joining phylogenetic tree of 14 *Puccinia graminis* f. sp. *tritici* pathotypes detected during 2016–2022.

One hundred seventy-five alleles were amplified by 37 *Puccinia* sp.-specific SSRs among 14 pathotypes. The number of alleles varied from two (PgSUN27, PGTG 00856, PGTG 07438, SSR-P AG-40, SSR-P CAAC- 44, and SSR-P TCCG-36) to eight (SSR-P CT-36, PtESSR22, PtESSR26, and PtESSR33) with an average of 4.8 alleles per locus. The heterozygosity of these SSRs ranged from 0.24 (SSR-P CAA-60) to 0.5 (PgSUN27 and PtESSR33). The heterozygosity of 21 SSRs was ≥48. The average polymorphic information index (PIC) value of these primers was 0.34 (**Supplementary Table 1**). An unweighted neighbor-joining (NJ) cluster analysis produced three distinct molecular groups (MG I to III). Seven and six pathotypes were grouped in MG I and II, respectively, while pathotype 42 with poor genetic similarity with the rest of the pathotypes formed a distinct MG III (**Figure 4**). The MG I and II were further divided into two subgroups (Ia, Ib; and IIa, IIb). Pathotypes 21, 21A-2, 21-1, and 40A were clustered in subgroup Ia, while pathotypes 40-2, 40-3, and 34-1 occupied subgroup Ib. Three pathotypes were clustered in subgroup IIa (117, 117-6, and 122) and subgroup IIb (11, 11A, and 15-1). Interestingly, pathotypes with nearly similar virulence patterns were grouped in one molecular group/subgroup. For example, pathotypes 21, 21A-2, and 21-1 in subgroup Ia, 11 and 11A in subgroup IIb, 117 and 117-6 in IIa, and 40A, 40-2, and 40-3 were grouped in MG I.

### Seedling response and postulation of stem rust resistance genes

The seedling stem rust response of 40 wheat varieties against 23 *Pgt* pathotypes indicated substantial resistance in these varieties (**Supplementary Table 3**). Pathotype 40-3 with compatible reactions in 15 varieties was the most virulent. Likewise, pathotypes 11, 24A, 40-2, 117-1, 117-2, and 117-6 were virulent on six varieties, while 117A-1 was virulent on five varieties and 40-1, 184-1, and 295 on four varieties (**Supplementary Table 3**). Fourteen varieties exhibited resistant response to all 23 pathotypes, including the stem rust resistance conferred by *Sr31* and *Sr24* in 10 and 2 varieties, respectively (**Table 4**). Two varieties (HI1628 and HI1655), exhibiting resistance to all pathotypes, had *Sr2*, which was postulated based on micro flecking, an *Sr2*-linked morphological marker and identified through genetic analysis. We could postulate 12 *Sr* genes (*Sr2*, *Sr11*, *Sr7b*, *Sr31*, *Sr8a*, *Sr5*, *Sr9b*, *Sr13*, *Sr24*, *Sr28*, *Sr30*, and *Sr8b*), individually or in combination, in 39 wheat varieties. No *Sr* gene was postulated in MACS4100 as its resistance response to 23 pathotypes did not match with any known *Sr* genes. The frequency of *Sr2* was highest and postulated in 67.5% of varieties. It was followed by *Sr11*, *Sr7b*, *Sr31*, and *Sr8a*, which were postulated in 32.5%, 30%, 25%, and 10% varieties, respectively. The *Sr* genes *Sr28*, *Sr30*, and *Sr8b* were postulated only in HI1621, VL2041, and KRL19 (**Table 4**), respectively.

**Table 4:** List of *Sr* genes postulated in 39 wheat varieties using gene matching technique.

Resistance/*Sr* genes	Number of varieties	Detail of varieties
*Sr31+5+*	1	HS507
*Sr31+2+*	6	HD2733, HD3043, HD3090, MACS6768, PBW771, VL907
*Sr31+*	3	HI1633,HI1634, HI1650
*Sr24+2+*	2	CG1029, MP3288
*Sr28+*	1	HI1621
*Sr30+5+11+*	1	VL2041
*Sr8b+9b+11+2+*	1	KRL19
*Sr8a+5+11+2+*	1	DBW252
*Sr8a+9b+11+*	1	HS562
*Sr8a+11+2+*	1	HD2967
*Sr8a+2+*	1	NIAW3170
*Sr9b+11+*	1	HD3118
*Sr13+2+*	1	HD3293
*Sr13+*	1	HI1654
*Sr11+7b+2+*	2	DDW47 (d), HD3171
*Sr11+2+*	4	HD3249, HI8498 (d), MP3336, NIDW1149 (d)
*Sr11+*	1	RAJ4083
*Sr7b+2+*	6	DDW48 (d), HI8826 (d), HI8830 (d), HPW349, KRL210, WH1124
*Sr7b+*	2	CG1036, HI1653
*Sr2+*R*	2	HI1628, HI1655

*Resistant to all pathotypes.

### Adult plant responses of wheat varieties against stem rust

The ACI, AUDPC, and apparent infection rate (*r*) of wheat varieties were calculated based on their screening at stem rust hot spot locations consecutively for 2 years (**Table 5**). There were substantial variations in stem rust severity among different varieties across the locations. According to the Wilcoxon signed-rank test, the top 10 best-performing wheat varieties had ≤3.72 ACI and ≤103.40 AUDPC scores. The ACI, AUDPC, and *r*-values of all wheat varieties from all five locations ranged from 1.12 (HI1655) to 38.28 (KRL210), from 33.60 (HI1655) to 1,203.40 (KRL210), and from 0.009 (HI1655 and HI8826) to 0.063 (KRL19), respectively. Based on ACI values, these wheat varieties were grouped into highly resistant (ACI ≤ 5), resistant (ACI >5 to 10), moderately resistant (ACI >10 to 15), and susceptible (ACI>15). Among 40 varieties, 18 were highly resistant, 12 were resistant, 5 were moderately resistant, and the remaining 5 were classified as susceptible to stem rust (**Table 5**). Contrary to test entries, the stem rust severity on Agra Local was very high (>40S) at all locations and crop seasons. It demonstrated 58.0 and 2,240.0 ACI and average AUDPC scores, respectively, which were significantly high from test varieties. Wheat variety HI1655, with the lowest AUDPC and “*r*” values was the most resistant among all varieties, while KRL210 was highly susceptible with high AUDPC and “*r*” values. HI1655 has shown immune response at multiple locations during both the years. Some durum cultivars, viz., HI8826 and HI8830, were also immune to stem rust at multiple locations. The cultivars with lower ACI values were also strong to moderate slow rusters as these had lower AUDPC values and infection rates. The KRL210 was the most vulnerable to stem rust with the highest ACI (38.28) and AUDPC (1203.4) values.

**Table 5:** Stem rust severity, coefficient of infection (ACI), area under disease progress curve (AUDPC), and apparent infection rate (*r*) of wheat varieties arranged in increasing Wilcoxon signed-rank; and detail of *Sr* genes identified in these varieties using molecular markers.

Variety	Final Disease Score										ACI	Average AUDPC	Average *r*	Rank	*Sr* gene
	Niphad		Indore		Dharwad		Pune		Powarkheda						
	20-21	21-22	20-21	21-22	20-21	21-22	20-21	21-22	20-21	21-22					
HI1655	0	0	5R	5MR	10MS	0	TR	0	0	0	1.12	33.60	0.009	1	*Sr2*
HI8826 (d)	0	0	5MR	5MR	10MS	0	TMR	0	0	TR	1.26	37.60	0.009	2	*Sr57*
HI1654	0	0	5MS	5MR	10S	0	TR	0	0	0	1.62	48.60	0.010	3	*Sr38, Sr2*
HI8830 (d)	0	0	TMS	5R	10MS	0	TR	0	10MS	0	1.80	48.80	0.025	4	*Sr57*, *Sr58*
CG1036	0	0	10MR	5MS	0	0	10MS	0	0	0	1.60	54.00	0.014	5	*Sr24*
HI1633	0	10MR	10MR	5MR	10MS	0	5R	0	0	TR	1.92	57.40	0.012	6	*Sr57*, *Sr31, Sr2*
HI1650	0	TMR	10MR	5MR	10MS	TMS	10MS	0	0	0	2.32	69.60	0.019	7	*Sr57*, *Sr31, Sr2*
VL907	0	0	20MR	5MR	5MS	0	15MS	0	TR	TR	2.64	78.80	0.022	8	*Sr57*
HD2733	0	0	10MS	10MR	10MS	0	5MR	0	20MR	10MR	3.40	94.40	0.033	9	–
HS507	0	15MS	20MR	10MR	5MS	TMS	TR	0	10MS	TR	3.72	103.40	0.034	10	*Sr38, Sr2*
MACS6768	0	5MR	10MR	5R	10S	0	20MS	0	0	20MR	4.10	115.40	0.033	11	*Sr31, Sr2*
VL2041	0	5MS	20S	5S	0	0	10S	TS	0	0	4.00	120.00	0.020	12	*Sr2*
NIAW3170	0	0	40MR	10MR	10MS	5MS	10MR	0	TR	TR	3.64	120.80	0.023	13	*Sr2*
PBW771	0	0	20MS	10MR	10MS	0	15MR	0	10MR	0	3.80	122.40	0.035	14	–
HD2967	0	0	20MS	10MR	20S	0	TR	0	5MR	0	4.22	130.60	0.020	15	*Sr38*
HD3090	0	15MS	20MR	10MS	10S	0	20MR	0	TR	TR	4.64	144.80	0.030	16	*Sr24, Sr31, Sr2*
CG1029	R	5MS	10MR	10MR	5MS	0	10MR	20S	10MS	0	4.82	146.00	0.048	17	*Sr31, Sr2*
MP3288	0	10MS	20MR	10MR	5MS	0	10MR	0	TR	20S	4.82	150.40	0.024	18	*Sr24*
DDW47(d)	0	5MS	10MR	30MS	10S	0	5MR	0	10MR	20MR	5.60	156.00	0.035	19	*Sr58*
HD3043	0	5MR	40MR	10MR	10MS	5MS	20S	0	0	TR	5.42	162.40	0.031	20	*Sr38, Sr2*
NIDW1149 (d)	0	20MS	10MR	20MS	5MS	0	TR	0	20MS	TR	6.04	181.00	0.029	21	–
RAJ4083	0	15MS	10MR	5S	10S	TMR	30S	0	0	0	6.14	193.20	0.026	22	*Sr57, Sr2*
MACS4100 (d)	0	0	20S	20MS	10MS	0	20MS	0	10MR	TR	6.42	200.40	0.033	23	*Sr57*
HI1628	R	15MS	30MS	10MS	10MS	TMR	15MR	0	0	20MR	6.66	211.80	0.028	24	*Sr2*
HPW349	0	5MS	30MS	20S	5MS	0	10MS	0	20MS	TR	7.62	218.80	0.052	25	*Sr57, Sr2*
HD3249	0	10MR	10MS	10S	10MS	0	40S	0	0	20MR	7.80	238.40	0.045	26	*Sr38, Sr2*
HI1634	0	5MR	20MR	5R	10MS	0	60S	0	TR	TR	7.94	243.80	0.021	27	*Sr31, Sr2*
HI1621	5S	20MR	20MS	40MR	10MS	5MS	20S	0	10MR	TR	8.12	263.80	0.049	28	*Sr2*
DDW48 (d)	0	5MS	20MS	20MS	20S	0	20MR	0	40MS	10MR	10.00	286.00	0.045	29	*Sr57*
MP3336	0	10S	20S	10MS	10MS	0	20MS	0	0	40MS	9.40	314.00	0.034	30	*Sr2*
HD3171	0	20S	NG	40S	10MS	NG	10MS	0	0	40MS	14.00	395.00	0.035	31	*Sr58, Sr38*
HI8498 (d)	0	10S	20S	30MS	5MS	0	30S	0	20MS	40MS	13.60	398.40	0.044	32	–
DBW252	0	20MS	40MS	20S	20S	TMS	40S	0	0	TR	12.90	398.80	0.021	33	*Sr38*
KRL19	5MR	0	NG	10MS	10S	5MS	80S	10S	10MR	20MR	14.00	424.89	0.063	34	–
HI1653	10MS	20MS	20MS	20MS	10MS	TMS	60S	0	0	20MR	13.28	450.80	0.044	35	*Sr38, Sr2*
WH1124	0	10S	20MS	40S	5MS	5MS	20MS	30S	0	40MS	15.20	464.00	0.041	36	*Sr2*
HS562	20S	40S	40S	20S	5MS	0	40S	40S	0	10S	21.40	650.40	0.059	37	*Sr2*
HD3293	10S	20S	60S	30S	10MS	TMR	40S	30S	10MS	20S	22.64	691.60	0.055	38	*Sr2*
HD3118	5MS	15MS	60S	20S	10MS	0	60S	40S	40S	40S	28.40	947.00	0.053	39	*Sr2*
KRL210	15MS	40S	40S	40S	20S	TMS	80S	70S	40S	40S	38.28	1,203.40	0.060	40	*Sr2*
Agra Local (Susceptible Check)	40S	80S	80S	80S	40S	60S	60S	60S	40S	40S	58.00	2,240.00	0.043	41	–

### Identification of *Sr* genes using molecular markers

Genetic analysis of 40 wheat varieties using six molecular markers closely linked to selected *Sr* genes indicated the presence of six *Sr* genes (*Sr2*, *Sr57*, *Sr58*, *Sr24*, *Sr31*, and *Sr38*) in 35 varieties. The APR gene *Sr55* was not identified in any of the varieties with the CFD71 marker. *Sr2* gene linked marker (Xgwm533) indicated the presence of this gene in 24 varieties (**Supplementary Figure 1**). Other APR genes, i.e., *Sr58* and *Sr57*, were confirmed in three and nine varieties, respectively (**Table 5**), while the ASR genes *Sr24*, *Sr31*, and *Sr38* were identified in three, six, and eight varieties, respectively. A combination of two/three *Sr* genes was marked in CG1029 (*Sr31* and *Sr2*), HD3043 (*Sr38* and *Sr2*), HD3090 (*Sr24*, *Sr31*, and *Sr2*), HD3171 (*Sr58* and *Sr38*), HD3249 (*Sr38* and *Sr2*), HI1633 (*Sr57, Sr31*, and *Sr2*), HI1634 (*Sr31* and *Sr2*), HI1650 (*Sr57*, *Sr31*, and *Sr2*), HI1653 (*Sr38* and *Sr2*), HI1654 (*Sr38* and *Sr2*), HI8830 (*Sr57* and *Sr58*), HPW349 (*Sr57* and *Sr2*), HS507 (*Sr38* and *Sr2*), MACS6768 (*Sr31* and *Sr2*), and RAJ4083 (*Sr57* and *Sr2*).

## Discussion

Wheat stem rust has re-emerged as a serious menace to wheat production globally since the evolution of Ug99 in Uganda. To date, about 15 variants in the Ug99 lineage have been identified within its lineage. Some of these variants have spread to several wheat-growing areas in different countries including Zimbabwe, South Africa, Sudan, Yemen, Egypt, and Iran. The Ug99 variants and other new races reported from East Africa (Digalu race; Olivera et al., 2015), Europe (Olivera Firpo et al., 2017; Lewis et al., 2018), and Central Asia (Shamanin et al., 2020) have caused severe localized stem rust outbreaks and endangered wheat productivity and food security (Lewis et al., 2018). The further spread of the races in the Ug99 lineage to major wheat-growing countries in Asia, including India where almost 7 million hectares area in Central and Southern India is highly prone to stem rust outbreaks, and beyond could devastate wheat production in the affected areas (Olivera et al., 2018). Therefore, survey for monitoring the evolution or introduction of *Pgt* virulences supported by virulence phenotypes and molecular genotypes is crucial and must be undertaken regularly. Moreover, replacing stem rust-susceptible wheat varieties with resistant ones is a systematic approach to minimize yield losses and thwart pathogen survival (Randhawa et al., 2018; Prasad et al., 2020b). The present study was designed to monitor the variability and distribution pattern of *Pgt* isolates in India and neighboring countries from 2016 to 2022 through exhaustive surveillance, virulence phenotyping, and SSR genotyping, evaluate recently released wheat varieties for stem rust response at seedling and adult plant stages during two crop seasons at five stem rust-prone geographically different areas, and to undertake genetic analysis of wheat varieties to identify *Sr* genes in them.

The occurrence of wheat stem rust was sporadic during the crop years 2016 to 2022. Stem rust incidence was extremely low during 2015–2016 and 2021–2022 as we could detect only a few isolates of pathotypes 11, 21-1, 21A-2, 40A, 40-2, 40-3, and 122 from 54 samples during 2015–2016, and 11, 21, 34-1, 40A, and 40-2 from 62 samples during 2021–2022 (**Table 3**). Most of the advanced wheat material was stem rust-free while some old genotypes and admixtures had up to 80S severity under natural conditions. The availability of *Sr31* and other known and unknown *Sr* genes in recently developed wheat varieties, effective against all known pathotypes of stem rust in India and neighboring countries, accompanied by poor rainfall and a significant reduction in relative humidity over the years could have hampered the survival and infection rate of the natural population of *Pgt* and, therefore, poor stem rust incidence (Ayliffe et al., 2008; Prank et al., 2019). As revealed previously (Prasad et al., 2018; Prasad et al., 2022), the *Pgt* population reported from India and Nepal remained avirulent to *Sr7a, Sr26, Sr27, Sr31, Sr32, Sr33, Sr39, Sr40, Sr43, SrTmp*, and *SrTt3* to date. Some of these genes, for instance, *Sr26*, have been found to be effective against the *Pgt* population including Ug99 globally. Hence, these genes can be targeted for prudent wheat breeding for stem rust resistance through their pyramiding with other effective major and minor genes (Singh et al., 2015). The *Sr26* has been extensively used in the Australian wheat breeding program. Still, its use in India is seldom and no Indian wheat variety, including the tested varieties in this study, is reported to contain it.

**Figure 5:**
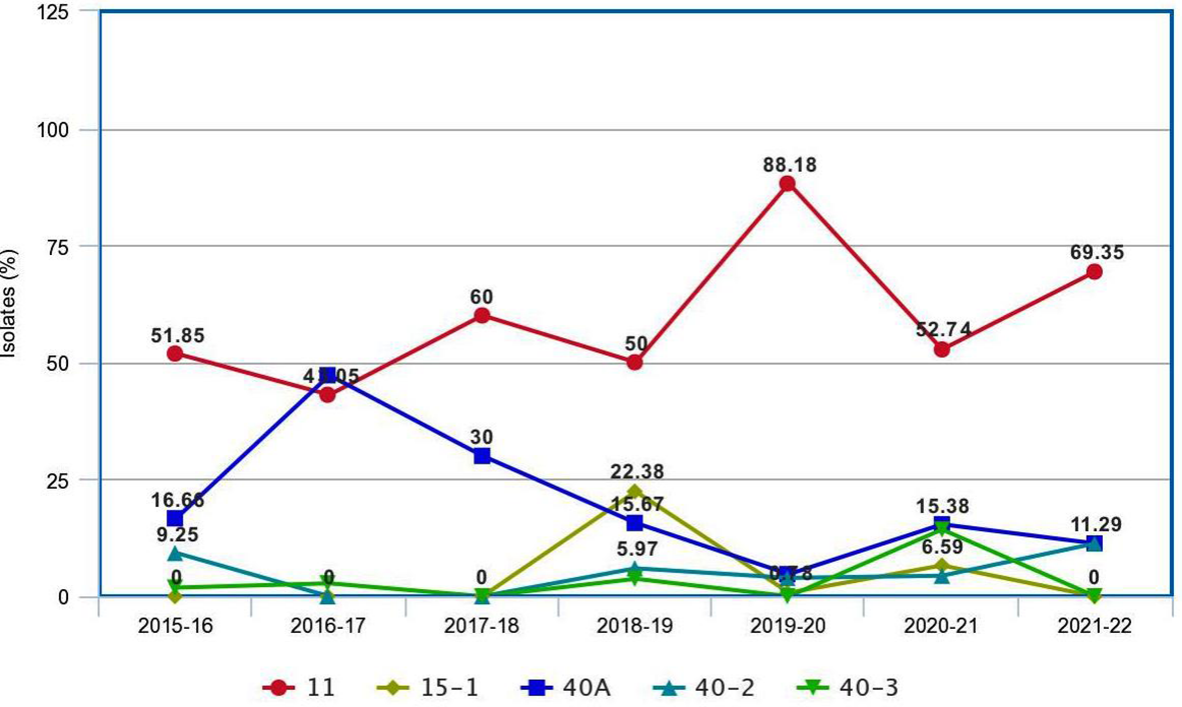
The pattern of occurrence of predominant pathotypes of *Puccinia graminis* f. sp. *tritici* on wheat in the Indian subcontinent during 2016–2020.

A comparison of *Pgt* pathotypes identified in the present study revealed some dissimilarity with previous reports (Jain et al., 2013; Prasad et al., 2018). Pathotypes 42, 117, and 117-6, which were not detected during 2009–2015, reappeared during this period, whereas pathotype 40-1, virulent on *Sr24*, was present during 2009–2015 but did not appear afterward (Prasad et al., 2018). Pathotypes 42 (2019–2020, Karnataka), 117 (2017–2018, Gujarat), and 117-6 (2017–2018, Karnataka) made their reappearance after a long span of more than 20, 19, and 9 years, respectively (Bhardwaj et al., 2003; Bhardwaj et al., 2006; Jain et al., 2013). The *Pgt* pathotypes in virulence group 21, earlier used to dominate northern India and parts of Nepal, were also detected from Gujarat, Madhya Pradesh, Karnataka, and Tamil Nadu (**Table 3**). Likewise, a primitive *Sr24* virulent pathotype 34-1, first identified from Madhya Pradesh in 1991 and prevalent on some local wheat landraces only in far north Leh Ladakh region afterwards, was identified again from Madhya Pradesh during 2021–2022. Such a shift in virulence pattern over time is not surprising, as the prevalence of some pathotypes in a particular crop season and their establishment in a new region depend on the diversity and resistance level of wheat varieties cultivated during that season (Singh, 1991), and to some extent on the prevailing environmental conditions such as temperature, rainfall, and relative humidity in particular (Roelfs et al., 1992). A similar shift in virulence pattern for stem rust of wheat has also been reported in many other countries including Canada, Mexico, the USA, Australia, and South Africa (Le Roux and Rijkenberg, 1987; Singh, 1991; Park and Wellings, 1992; Fetch, 2005; Jin, 2005; Terefe et al., 2016); for leaf (brown) rust from USA, Pakistan, India, and Egypt (Kolmer et al., 2017; Khadegah et al., 2019; Kolmer, 2019; Bhardwaj et al., 2021); and for stripe (yellow) rust from Canada, India, and other Asian countries (Hovmoller et al., 2011; Gangwar et al., 2022). There was a major shift in the frequency of predominant pathotypes of *Pgt* in India from 2016 to 2022. For instance, the frequency of pathotype 40A, which was the most predominant pathotype for almost three decades (Bhardwaj, 2012), reduced significantly after 2013–2014. Pathotype 11 remained most predominant since 2013–2014 throughout the current study period except during 2016–2017, when 40A surpassed pathotype 11 again (**Figure 5**). Although both pathotypes 40A and 11 are thought to have more virulence and better fitness potential over others, the modifications in the environmental conditions and altered varietal spectrum could have better-supported pathotype 11 over 40A during these years. Similar assumptions were made to justify the dominance of one pathotype over another in the case of sunflower rust caused by *Puccinia helianthi* Schw. (Prud'homme and Sackston, 1990), wheat leaf rust (Bhardwaj et al., 2014), and stripe rust (Hovmoller et al., 2011). The optimum temperature, sufficient moisture, and cultivation of diverse wheat genotypes supported maximum virulence diversity in *Pgt* populations during 2018–2019 and 2020–2021 (Prasad et al., 2019; Prasad et al., 2021c). Eight pathotypes with different virulence structures were identified during these two crop seasons in Gujarat, Himachal Pradesh, Karnataka, Madhya Pradesh, Maharashtra, Tamil Nadu, and Nepal.

The phylogenetic analysis of 14 *Pgt* pathotypes, using 37 *Puccinia* sp.-specific polymorphic SSRs, clustered them in three molecular groups (**Figure 4**), indicating the existence of very few lineages in the whole *Pgt* population. The heterozygosity and PIC values of these SSRs suggested that these markers had sturdy differentiating power as suggested by Shete et al. (2000) and Botstein et al. (1980). The molecular grouping of *Pgt* pathotypes, generated through unweighted neighbor-joining (NJ) cluster analysis in DARwin 6.0.14, corroborated, up to some extent, virulence-based grouping of these pathotypes, which was in agreement with our previous findings (Prasad et al., 2022). Pathotypes 21, 21-1, and 21A-2; 11 and 11A; 117 and 117-6; and 40A, 40-2, and 40-3, sharing almost matching avirulence/virulence structure as reported previously (Prasad et al., 2018; Prasad et al., 2022), were clustered in one MG/subgroup (**Figure 4**). Such an association of virulence phenotypes and SSR genotypes among *P. triticina* (Wang et al., 2010) and *P. striiformis tritici* (Hovmoller et al., 2002) isolates is also reported, which is not uncommon if the genes responsible for virulence/pathogenicity are extensively distributed in pathogen genome (Ordonez and Kolmer, 2009). The comprehensive evolutionary pattern of all these *Pgt* pathotypes and others reported from the Indian sub-continent is already discussed previously (Prasad et al., 2022). Lower genetic diversity among *Pgt* pathotypes may be attributed to the clonal multiplication and long-distance migratory nature of the pathogen in the Indian sub-continent (Wingen et al., 2013).

Stem rust response of 40 wheat varieties revealed substantial resistance in these varieties against 23 pathotypes of *Pgt* (**Supplementary Table 2**) at seedling and against 11, 21A-2, 40A, 117-6, 122, and other naturally occurring isolates at adult plant stages from five geographically different locations (**Supplementary Table 3**; **Table 5**). At the seedling stage, 14 varieties exhibited resistant response against all 23 pathotypes, which included resistance conferred by *Sr31* in 10 varieties and by *Sr24* in 2 varieties. Despite the fact that 2 (34-1 and 40-1) of the 23 pathotypes are particularly virulent on *Sr24* (Jain et al., 2013), these pathotypes were unable to establish a compatible relationship with two *Sr24*-containing wheat varieties (CG1029 and MP3288). Such interactions could result from the presence of some additional unknown effective *Sr* gene other than *Sr24*, which might impede pathotypes 34-1 and 40-1 from establishing compatible interactions with these varieties (Dakouri et al., 2013). The Indian population of *Pgt* possesses very low virulence frequencies on *Sr31*, *Sr24*, and *Sr30* (Prasad et al., 2022), which are among 12 *Sr* genes postulated in 39 varieties (**Table 4**), and therefore, these varieties could be targeted for skillful gene deployment. Generally, *Sr2, Sr5, Sr6, Sr7a, Sr7b, Sr8a, Sr8b, Sr9b, Sr9e, Sr11, Sr12, Sr13, Sr17, Sr21, Sr24, Sr25, Sr30*, and *Sr31* are frequently occurring *Sr* genes in Indian wheat varieties. Among these, *Sr7b, Sr9e, Sr11*, and *Sr13* are more common in durum wheat, while *Sr2, Sr11*, and *Sr31* are mostly postulated in bread wheat (Jain et al., 2013). The frequency of *Sr2*, postulated in 67.5% of varieties based on micro flecking: an *Sr2*-linked morphological marker (Khan et al., 2017), was the highest. The *Sr2*, along with other slow rusting *Sr* genes such as *Sr55, Sr56, Sr57*, and *Sr58*, could be a valuable resource to have and utilize in combination with other effective major genes, i.e., *Sr26*, for achieving durable stem rust resistance (Singh et al., 2015).

The slow rusting/APR/field response of 40 wheat varieties and a susceptible check (Agra Local) were assessed based on the valuation of the final disease score (FDS), ACI, AUDPC, and apparent infection rate. The findings showed that the tested wheat varieties had more available resistance than Agra Local, which was severely infected during both the crop years at all five locations. Generally, the slow rusting behavior in wheat has been estimated and reported through assessment of disease severity, AUDPC, CI values, and apparent infection rates (Broers et al., 1996; Pathan and Park, 2006). The FDS is also used as a parameter to assess the slow rusting character of wheat genotypes by many earlier researchers (Ali et al., 2009); however, ACI, AUDPC, and apparent infection rate are considered most appropriate and preferred. Among 40 test varieties, 30 had ACI values ≤ 17.24% and 23 had AUDPC values ≤ 8.94% of the check (**Table 5**), which specify high to moderate levels of slow rusting resistance character of these varieties (Ali et al., 2009). Furthermore, most of the varieties had MR/MS-type infection responses, which are the characteristics of the slow rusting genes, conferring partial field resistance (Parlevliet, 1988). The Wilcoxon signed-rank test categorized HI1655, HI8826, HI1654, HI8830, CG1036, HI1633, HI1650, VL907, HD2733, and HS507, with ≤3.72 ACI and ≤103.40 AUDPC scores, as the 10 best-performing varieties for stem rust APR. Among these, the *Sr31* was postulated in HI1633, HI1650, VL907, HD2733, and HS507, based on their response to 23 *Pgt* pathotypes at the seedling stage and its linkage to *Lr26*/*Yr9*. Lower ACI and AUDPC values in these five varieties are implicit since *Sr31* virulences are not reported from South Asian countries (Bhardwaj et al., 2019). Although *Sr31* lost its effectiveness in East Africa and the Middle East due to Ug99 and other variants in its lineage, yet it is valued plus owing to its contribution to yield advantage and effectiveness to stem rust and other diseases in many countries including those in South Asia (Cox et al., 1995; Yu et al., 2014). Some weak stem rust resistance genes, i.e., *Sr13+*, *Sr7b+2+*, *Sr7b+*, and *Sr2+*, were postulated in the remaining (5) top 10 varieties HI1654, HI8826 (d) and HI8830 (d), CG1036, and HI1655, respectively. The genetic analysis of wheat germplasm using rust resistance gene linked molecular markers is another substitute to traditional gene postulation and could help in the swift and precise identification of resistance genes (Goutam et al., 2013). The molecular markers closely linked to rust resistance genes have frequently been identified and utilized in marker-assisted breeding for rust resistance (Mago et al., 2013). Besides being quick and unambiguous, this method could also circumvent the problems associated with traditional gene postulation such as plant stage-dependent gene expression, temperature, and other abiotic factor-dependent gene interactions. In the present study, genetic analysis of wheat varieties using molecular markers closely linked to *Sr31* and *Sr24* confirmed the gene postulation results in the majority of the cases except for *Sr24* in the wheat variety CG1036 and *Sr31* in CG1029. The gene postulation using the gene matching technique listed the presence of *Sr7b* and *Sr24* in CG1036 and CG1029, respectively (**Table 4**). None of the six *Sr* genes could be identified in five varieties (HD2733, PBW771, NIDW1149, HI8498, and KRL19) through genetic analysis, indicating the presence of some other known or unknown ASR/APR genes. All these markers have been extensively used to identify *Sr* genes or *Yr* and *Lr* genes linked to *Sr* genes. For instance, marker iag95 was effectively used to identify *Sr31* in some Chinese wheat cultivars (Xu et al., 2017; Xu et al., 2018). Similarly, *Sr38* linked to *Yr17* and *Lr37* was reported from wheat cultivars in China and Nebraska using VENTRIUP-LN2, an *Sr38*/*Yr17/Lr37* linked marker (Peng et al., 2013; Xue et al., 2014; Xu et al., 2017; Mourad et al., 2019). Yang et al. (2008) and Alma et al. (2012) identified the *Sr57* gene in some Chinese and Central Asian wheat cultivars, respectively, using the *csLV34* marker. The WMC44 marker was applied for the molecular analysis of slow rusting resistance gene *Sr58/Lr46/Yr29* in triticale cultivars (Skowrońska et al., 2020). Herrera-Foessel et al. (2014) used CFD71 marker to detect *Sr55* in wheat varieties that conferred APR to stem rust and powdery mildew in wheat. The marker Sr24#50 was used to evaluate and identify *Sr24* in wheat lines from Gansu province in China (Xu et al., 2017). This marker indicated the presence of *Sr24* in CG1036 contrary to gene postulation, which indicated the presence of a relatively weak *Sr7b* in the same variety (**Tables 4**, **5**). The susceptibility of CG1036 to several *Pgt* pathotypes, other than *Sr24* virulences (34-1 and 40-1, **Supplementary Table 3**), also ruled out the presence of *Sr24* in it (Prasad et al., 2018). The SSR marker Xgwm533 has been extensively used for the identification of *Sr2*/*Yr30* in wheat worldwide (Mago et al., 2011; Khan et al., 2017; Rahmatov et al., 2019). We could identify *Sr2* in 24 wheat varieties using this marker. The *Sr2* was confirmed in 13 varieties using both gene postulation and marker analysis approaches. However, when looking at specific methods individually, it was identified in 11 varieties through marker analysis and in 14 varieties through gene postulation (**Tables 4**, **5** and **Supplementary Figure 1**). Similar observations were reported previously (Khan et al., 2017). The failure of the Xgwm533 marker to validate the micro- flecking-based postulation of *Sr2* in some varieties could be justified by the fact that many of these varieties might lack *Sr2* and micro-flecking could have occurred due to external environmental factors influencing host–pathogen interactions (Mago et al., 2011). Another probability could be that the variety with micro-flecking might have separated from *Sr2* or that the marker was not diagnostic (Rahmatov et al., 2019). Similar observations were reported for *Lr34*/*Yr18*/*Pm38* linked to more accurate molecular markers, where markers reported the presence of these genes even in susceptible wheats (Lagudah et al., 2009). The collective gene postulation and marker analysis results indicated that wheat varieties HI1655 and HI1654 might harbor some effective known/unknown *Sr* genes other than *Sr2* to confer adequate APR. Therefore, these varieties could be appraised for durable stem rust resistance breeding in wheat. More importantly, further genetic analysis of these varieties could reveal some interesting loci that confer resistance to all Indian *Pgt* pathotypes. The locus contributing to stem rust APR, which includes the *Sr2* gene, has been found to be effective and recommended for use in breeding programs (Rutkoski et al., 2014; Singh et al., 2015). The deployment of *Sr2* is largely advised in combination with other effective *Sr* genes as it (alone) could not offer adequate protection under epidemic situations (Haile and Roder, 2013).

In conclusion, 14 *Pgt* pathotypes were identified as a result of surveys on the prevalence of wheat stem rust from 2016 to 2022 and pathotype analysis of the samples gathered during the surveys. Pathotypes 11 and 40A remained the most predominant from the previous reports, while pathotypes 42, 117, and 117-6 made their reappearance after more than 9 years. Remarkable stem rust resistance, conferred by some effective known ASR (*Sr24*, *Sr31* and *Sr38*) and APR (*Sr2, Sr57*, and *Sr58*) genes, as revealed by gene postulation and genetic analysis using closely linked markers to these genes, was observed among most wheat varieties. The values of adult plant slow rusting parameters in these varieties were highly encouraging and could prove useful for stem rust management through their deployment or as durable stem rust resistance sources for future breeding program. These findings would be useful in deploying appropriate resistant varieties and streamlining wheat breeding approaches to combat stem rust. These conclusions provide good opportunity to explore some novel resistances where already known slow resisting loci are not identified. In view of the dynamic nature, virulence diversity, and long-distance migration of *Pgt* isolates, their monitoring the shifting virulence pattern, searching for new sources of resistance, and their transfer into high-yielding cultivars must be sustained to mitigate the impact of stem rust menace on wheat.

## Data availability statement

The original contributions presented in the study are included in the article/**Supplementary Material**. Further inquiries can be directed to the corresponding author.

## Author contributions

PP and SB conceived the experiment. PP, RT, SS, OG, CL, SA, SuK, SoK, and AM conducted the lab and greenhouse experiments. TP, SN, GH, BG, and KM conducted field experiments. PP, HK, VG, CM, and StK conducted statistical analysis. PP wrote the first draft of the article. SB, SudK, and GS edited and improved the article. All authors read the final version of the manuscript and approved it.
