# Supplementary material for: Virulence and genetic analysis of Puccinia graminis tritici in the Indian sub-continent from 2016 to 2022 and evaluation of wheat varieties for stem rust resistance
Source: Front Plant Sci. 2023 Jul 14;14:1196808. doi: 10.3389/fpls.2023.1196808 (PMC10376725; doi:10.3389/fpls.2023.1196808)
Supplement: Supplementary file 1 [file DataSheet_1.docx]

**Supplementary Table 1. Primer sequences, annealing temperature (^0^C), Heterozygosity, and polymorphic information content (PIC) values of the simple sequence repeat primers used in the study**

| **S. No.** | **Marker/Locus** | **Primers (5’- 3’)** | **AT* (^0^C)** | **Heterozygosity** | **PIC**** | **Reference** |
| --- | --- | --- | --- | --- | --- | --- |
| **1** | PgSUN27 | F: TCAGCCCATCATCAGGACTC | 54 | 0.5 | 0.37 | Karaoglu et al. (2013) |
|  |  | R: CCTCCAGCCCAGTTCAGAGC |  |  |  |  |
| **2** | PGTG 03066 | F: CGAAAGAAAGGAAACGAAGGT | 50 | 0.4 | 0.32 | Prasad et al. (2018) |
|  |  | R: ACATCAATCTCGACCAATCTCC |  |  |  |  |
| **3** | PGTG 04483 | F: TCCCATCACATGCAGTAGTAGC | 48 | 0.45 | 0.35 |  |
|  |  | R: AATCTAATTGACAGCCTTGCGT |  |  |  |  |
| **4** | PGTG 00856 | F: ACAACAACAACAGCAGGACATC | 49 | 0.49 | 0.37 |  |
|  |  | R: TCGTTGAGGATGATTGAGTTTG |  |  |  |  |
| **5** | PGTG 07438 | F: ACTGGCTCATCATCATCTTCCT | 52 | 0.49 | 0.37 |  |
|  |  | R: CCAACCATTCCGACCTAATAAA |  |  |  |  |
| **6** | SSR-P GT-42 | F: GGGGTGAGTTTCTGTATTGA | 50 | 0.38 | 0.3 | Prasad et al. (2017a) |
|  |  | R: CAGAGATCATCGAGGAAAAC |  |  |  |  |
| **7** | SSR-P AG-40 | F: CTTTCTTACCCCCACAACTAC | 51 | 0.43 | 0.34 |  |
|  |  | R: CTCTCTCTCTCTCTCTCTCTCTC |  |  |  |  |
| **8** | SSR-P CT-36 | F: ACTCTCAAACTCACTCCCTCT | 48 | 0.48 | 0.36 |  |
|  |  | R: GACTACACCATTTCAAACCAA |  |  |  |  |
| **9** | SSR-P AC-32 | F: ACAAAACAAACAGATCCACTG | 49 | 0.39 | 0.31 |  |
|  |  | R: ACGTATTTGGTCTTCTTCTCC |  |  |  |  |
| **10** | SSR-P CAA-60 | F: AACTGCGAGGACAACTTTC | 52 | 0.24 | 0.21 |  |
|  |  | R: CGTCTGCTGAGTTTCTGTATT |  |  |  |  |
| **11** | SSR-P GGT-45 | F: GCTGCTTGATGGAGGATG | 55 | 0.48 | 0.36 |  |
|  |  | R: AACAGCTTCAGCGACCTC |  |  |  |  |
| **12** | SSR-P GTT-45 | F: GATGAGGTTGTTGAAGGAGA | 49.6 | 0.43 | 0.34 |  |
|  |  | R: ACCAGAACCAACAAAACAAC |  |  |  |  |
| **13** | SSR-P CAC-45 | F: GAAGACCATCCTCACGACT | 51 | 0.48 | 0.36 |  |
|  |  | R: TTCTTCTTGTTGGTTTTTCTG |  |  |  |  |
| **14** | SSR-P CAAC- 44 | F: AGCGTAGAGTCAGTCAGTCAG | 51 | 0.48 | 0.36 |  |
|  |  | R: GCTAATAAGGAGATTGGGTTG |  |  |  |  |
| **15** | SSR-P TATC-40 | F: AAGCGTGATCAAGTAGGTTTA | 50.4 | 0.3 | 0.26 |  |
|  |  | R: GATGGACAAGTAGAGAGATGG |  |  |  |  |
| **16** | SSR-P TCCG-36 | F: TTTTTCTAGATCCACCAACC | 50.4 | 0.47 | 0.36 |  |
|  |  | R: TACGAACAGGAGTCCCTCA |  |  |  |  |
| **17** | SSR-P TATTG-60 | F: TCAAACAACTTCATCCTGAAC | 48 | 0.49 | 0.37 |  |
|  |  | R: ATGTGATATCTTTTGGATTGG |  |  |  |  |
| **18** | SSR-P TCTTT-50 | F: GGGTTTATATGGTGGGTGT | 48 | 0.49 | 0.37 |  |
|  |  | R: GTTGAGTGGGTGAGATGAGTA |  |  |  |  |
| **19** | SSR-P ACAAAC-48 | F: ATACATTTTGGTTACCCACCT | 48.9 | 0.48 | 0.36 |  |
|  |  | R: TGTGTTTGTTTGTGTTTGTGT |  |  |  |  |
| **20** | SSR-P GCTGTT-60 | F: GATGAGCAGCATGAGGAG | 51.9 | 0.48 | 0.36 |  |
|  |  | R: CACCAGAACAACATACTCCAT |  |  |  |  |
| **21** | PtESSR6 | F: ATGATGTCCCGCTCACCT | 52 | 0.46 | 0.35 | Savadi et al. (2020) |
|  |  | R: ATCACAGAGTTGGCGATATG |  |  |  |  |
| **22** | PtESSR17 | F: CAAACTGCCCAATCTTTATCT | 53 | 0.48 | 0.36 |  |
|  |  | R: GTGCGAGCCTGTCCCTTC |  |  |  |  |
| **23** | PtESSR18 | F: CTCTGCCCCTCTCTCTCC | 50 | 0.49 | 0.37 |  |
|  |  | R: CTACCTCATCAGGCACCTT |  |  |  |  |
| **24** | PtESSR22 | F: ACAGAGGGAGCTCCACAA | 51 | 0.47 | 0.36 |  |
|  |  | R: CTCCCGCTACCCTTTCTC |  |  |  |  |
| **25** | PtESSR24 | F: CGTAGACGTTCACCTCGT | 49 | 0.48 | 0.36 |  |
|  |  | R: GGCGGTTACTGTTTTGTTT |  |  |  |  |
| **26** | PtESSR25 | F: TCTCGACGATCTGGACAT | 50 | 0.45 | 0.35 |  |
|  |  | R: GAGGTCGAGGACGAGGAC |  |  |  |  |
| **27** | PtESSR26 | F: AGGGAGGAGGATGATGGT | 55 | 0.49 | 0.37 |  |
|  |  | R: TGGAGGAGAAAGGATGAAC |  |  |  |  |
| **28** | PtESSR27 | F: GGATGAGAGATACAACAACCA | 53 | 0.47 | 0.36 |  |
|  |  | R: AACATTTGGGTGCAGTAAATA |  |  |  |  |
| **29** | PtESSR28 | F: ATTGTGGCGGCGGAGGAG | 54 | 0.49 | 0.37 |  |
|  |  | R: GATCTTGGACACCGAGAAG |  |  |  |  |
| **30** | PtESSR30 | F: GGACTTGCGTTCTACTACAAA | 53 | 0.49 | 0.37 |  |
|  |  | R: TACTCCACTTTTTAGCCTCCT |  |  |  |  |
| **31** | PtESSR31 | F: TCTCGAGGATCTCTAGGTAGC | 53 | 0.48 | 0.36 |  |
|  |  | R: GACGAGACCTCCGTATCC |  |  |  |  |
| **32** | PtESSR33 | F: AGTGACACCATGAATGAAAAA | 53 | 0.5 | 0.37 |  |
|  |  | R: CAAGAAAACAAAAACAGCACT |  |  |  |  |
| **33** | PtESSR34 | F: CATATGAAGACAGGGAGCAC | 54 | 0.36 | 0.29 |  |
|  |  | R: GTCATGGTGGATTGATTGA |  |  |  |  |
| **34** | PtESSR35 | F: GATTCCGGATTAGCCACTA | 53 | 0.47 | 0.36 |  |
|  |  | R: AAATAAGCAGCTCCCAATC |  |  |  |  |
| **35** | PtESSR36 | F: CTGTTTCTTGGTGATCAGGT | 54 | 0.46 | 0.35 |  |
|  |  | R: CCAGAACAGTCATCCTCCT |  |  |  |  |
| **36** | PtESSR38 | F: CTTGCTGTGCCGGTCCTT | 53 | 0.49 | 0.37 |  |
|  |  | R: CCTCTCCACCACCATGAC |  |  |  |  |
| **37** | PtESSR46 | F: TCCCAGAGTATGTGTTTTGTT | 53 | 0.49 | 0.37 |  |
|  |  | R: CGTGAGTTATGGATGGATG |  |  |  |  |
| *AT: Annealing temperature; **PIC: polymorphic information content | | | | | | |

**Supplementary Table 2. Detail of *Puccinia graminis tritici* pathotypes used for screening seedling stem rust response of wheat varieties and postulate *Sr* genes in them**

| **S. No.** | **Designation** | | | **Avirulence/virulence formula** |
| --- | --- | --- | --- | --- |
|  | **New** | **Old** | **North American**  **Equivalent*** |  |
| 1 | 79G31 | 11 | RRTSF | *Sr7a, 8a, 8b, 9e, 22, 23, 24, 25, 26, 27, 31, 32, 33, 35, 37, 39, 40, 43, Tmp, Tt3*/ *5, 6, 7b 9a, 9b, 9c, 9d, 9f, 9g, 10, 11, 13, 14, 15, 16, 17, 18, 19, 20, 21, 28, 29, 30, 34, 36, 38, McN* |
| 2 | 203G15 | 11A | RHTSF | *Sr7a, 8a, 8b, 9e, 11, 22, 23, 24, 25, 26, 27, 31, 32, 33, 35, 39, 40, 43, Tmp, Tt3*/*5, 6, 7b 9a, 9b, 9c, 9d, 9f, 9g, 10, 12, 13, 14, 15, 16, 17, 18, 19, 20, 21, 28, 29, 30, 34, 36, 37, 38, McN* |
| 3 | 123G15 | 15-1 | TKTSF | *Sr7a, 11, 24, 25, 26, 27, 31, 32, 33, 35, 37, 39, 40, 43, Gt, Tmp, Tt3*/*5, 6, 7b, 8a, 8b, 9a, 9b, 9d, 9e, 9f, 9g, 10, 12, 13, 14, 15, 16, 17, 18, 19, 20, 21, 22, 23, 28, 29, 30, 34, 36, 38, 42, 44, McN, Wld* |
| 4 | 9G5 | 21 | CHMQC | *Sr5, 7a, 8a, 8b, 9b, 9c, 9e, 10, 11, 12, 15, 21, 22, 23, 24, 25, 26, 27, 29, 30, 31, 32, 33, 34, 35, 37, 38, 39, 40, 43, Gt, Tmp, Tt3*/*6,7b, 9d, 9f, 9g, 13, 14, 16, 17, 19, 28, 36, McN* |
| 5 | 75G5 | 21A-2 | CCTJC | *Sr5, 6, 7a, 8a, 8b, 9a, 9c, 9e, 11, 12, 21, 22, 23, 24, 25, 26, 27, 29, 31, 32, 33, 35, 37, 38, 39, 40, 43, Gt, Tmp, Tt3*/*7b, 9b, 9d, 9f, 9g, 10, 13, 14, 15, 16, 17, 19, 28, 30, 34, 36, McN* |
| 6 | 5G19 | 24A | HRMSF | *Sr5, 7a, 8a, 8b, 9b, 9e, 24, 25, 26, 27, 28, 30, 31, 32, 33, 35, 36, 37, 39, 40, 43 , Tmp*/*2, 6, 7b, 9a, 9d, 9f, 9g, 10, 11, 12, 13, 14, 15, 16, 17, 18, 19, 20, 21, 22, 23, 29, 34, 36, 38, 42, 44, McN* |
| 7 | 10G13 | 34-1 | MCGGP | *Sr6, 7a, 8a, 8b, 9a, 9e, 10, 11, 13, 17, 19, 21, 22, 23, 24, 25, 26, 27, 30, 31, 32, 33, 35, 36, 37, 39, 40,43, Tmp, Tt3*/*5, 7b, 9b, 9d, 9f, 9g, 14, 15, 16, 18, 20, 28, 29, 34, 38, McN* |
| 8 | 62G29 | 40A | PTHSC | *Sr7a, 13, 21, 22, 24, 25, 26, 27, 30, 31, 32, 33, 35, 36, 37, 38, 39, 40, 43, Tmp, Tt3*/*5, 6, 7b, 8a, 8b, 9a, 9b, 9d, 9e, 9f, 9g, 10, 11, 14, 15, 16, 17, 18, 19, 20, 23, 28, 29, 34, McN* |
| 9 | 62G29-1 | 40-1 | PTHSM | *Sr7a, 13, 21, 22, 25, 26, 27, 30, 31, 32, 33, 35, 36, 37, 38, 39, 40, 43, Tmp, Tt3*/*5, 6, 7b, 8a, 8b, 9a, 9b, 9d, 9e, 9f, 9g, 10, 11, 14, 15, 16, 17, 18, 19, 20, 23, 24, 28, 29, 34, McN* |
| 10 | 58G13-3 | 40-2 | PKRSC | *Sr7a, 11, 13, 14, 21, 22, 23, 24, 26, 27, 29, 30, 31, 32, 33, 35, 37, 38, 39, 40, 43, Tmp*/*5, 6, 7b, 8a, 8b, 9a, 9b, 9d, 9e, 9f, 9g, 10, 12, 15, 16, 17, 18, 19, 20, 25, 28, 34, 36, 42, Wld-1, McN, Gt* |
| 11 | 127G29 | 40-3 | PTKSF | *Sr21, 22,24, 25, 26, 27, 31, 32, 33, 35, 36, 37, 39, 40, 42, 43, Tmp, Tt3*/*5, 6, 7a, 7b, 8a, 8b, 9a, 9b, 9d, 9e, 9f, 9g, 10, 11, 14, 15, 16, 17, 18, 19, 20, 23, 28, 29, 30, 34, 38, 44, McN, Gt* |
| 12 | 7G35 | 42B | HRHJC | *Sr2, 5, 8a, 8b,9a, 9c, 9e, 22, 24, 25, 26, 27, 28, 29, 30, 31, 32, 35, 36, 37, 38, 39, 40, 43, Tmp, Tt3* /*6, 7a, 7b, 9b, 9d, 9f, 10, 11, 12, 13, 14, 15, 16, 17, 19, 21, 23, 33, 34, McN* |
| 13 | 37G3 | 117 | KRCSC | *Sr7a, 5, 8, 9b, 12, 22, 24, 25, 26, 27, 28, 30, 31, 32, 33, 35, 36, 37/6, 7b, 9a, 9c, 9d, 9e, 9f, 10, 11, 13, 14, 15, 16, 17, 19, 21, 23, 29, 34* |
| 14 | 36G2 | 117A | KRCQC | *Sr5, 8, 9b, 10, 12, 13, 14, 16, 22, 24, 25, 26, 27, 28, 30, 31, 32, 33, 36, 37/6, 7b, 9a, 9c, 9d, 9e, 9f, 11, 15, 21, 23, 29, 34* |
| 15 | 38G18 | 117A-1 | HRHSC | *Sr5, 7b, 8, 12, 13, 22, 24, 25, 26, 27, 28, 30, 31, 32, 33, 35, 36, 37/6, 7a, 9a, 9b, 9c, 9d, 9e, 9f, 10, 11, 14, 15, 16, 17, 19, 21, 23, 29, 34* |
| 16 | 166G2 | 117-1 | JRHSC | *Sr5, 7a, 7b, 8, 12, 13, 14, 22, 24, 25, 26, 27, 28, 30, 31, 32, 33, 35, 36/6, 9a, 9b, 9c, 9d, 9e, 9f, 10, 11, 15, 16, 17, 19, 21, 23, 29, 34, 37* |
| 17 | 33G3 | 117-2 | KHCSC | *Sr5, 7a, 8a, 8b, 9b, 11, 12, 22, 24, 25, 26, 27, 28, 30, 31, 32, 33, 35, 36, 37, Tmp*/*2, 6, 7b, 9a, 9c, 9d, 9e, 9f, 9g, 10, 13, 14, 15, 16, 17, 19, 21, 23, 29, 34, McN* |
| 18 | 167G3 | 117-3 | KRCSC | *Sr5, 8a, 8b, 9b, 22, 24, 25, 26, 27, 28, 30, 31, 32, 33, 35, 36, 38 , 39, 40, 43, Tmp*/*2, 6, 7a, 7b, 9e, 9f, 9g, 10, 11, 12, 13, 14, 15, 16, 17, 19, 21, 23, 29, 34, 37, McN* |
| 19 | 37G19 | 117-6 | KRCSC | *Sr5, 8a, 8b, 9b, 22, 24, 25, 26, 27, 28, 30, 31, 32, 33, 35, 36, 37, Tmp*/*2, 6, 7a, 7b, 9e, 9f, 9g, 10, 11, 12, 13, 14, 15, 16, 17, 19, 21, 23, 29, 34, McN* |
| 20 | 7G11 | 122 | RRJQC | *Sr7a, 8a, 8b, 9e, 10, 12, 14, 15, 16, 17, 18, 19, 20, 22, 24, 25, 26, 27, 28, 30, 31, 32, 33, 35, 36, 37, 38, 39, 40, 43, Tmp, Tt3*/*5, 6, 7b, 9a, 9b, 9c, 9d, 9f, 9g, 11, 13, 21, 23, 29, 34, McN* |
| 21 | 53G1 | 184 | FPCSC | *Sr5, 6, 9b, 15, 18, 21, 22, 24, 25, 26, 27, 28, 29, 30, 31, 32, 33, 35, 36, 37, 38, 39, 40, 42, 43, Tmp, Tt3*/*7a, 7b, 8a, 8b, 9a, 9c, 9d, 9e, 9f, 9g, 10, 11, 12, 13, 14, 16, 17, 19, 20, 23, 34, McN* |
| 22 | 55G1 | 184-1 | FPHSC | *Sr5, 6, 15, 18, 21, 22, 24, 25, 26, 27, 28, 29, 30, 31, 32, 33, 35, 36, 37, 38, 39, 40, 42, 43, Tmp, Tt3*/*7a, 7b, 8a, 8b, 9a, 9b, 9c, 9d, 9e, 9f, 9g, 10, 11, 12, 13, 14, 16, 17, 19, 20, 23, 34, McN* |
| 23 | 7G43 | 295 | RRHQC | *Sr8a, 8b, 9e, 9c, 10, 12, 14, 15, 16, 18, 19, 20, 22, 24, 25, 26, 27, 28, 30, 31, 32, 33, 35, 36, 37, 38, 39, 40, 43, Tmp, Tt3*/*6, 5, 7a, 7b, 9a, 9b, 9d, 9f, 9g, 11, 13, 17, 21, 23, 29, 34, McN* |

**Supplementary Table 3. Seedling response (infection types) of 40 wheat varieties against 23 pathotypes of *Puccinia graminis tritici* and *Sr* genes postulated in these varieties.**

|  |  | **PATHOTYPES** | | | | | | | | | | | | | | | | | | | | | | |  |
| --- | --- | --- | --- | --- | --- | --- | --- | --- | --- | --- | --- | --- | --- | --- | --- | --- | --- | --- | --- | --- | --- | --- | --- | --- | --- |
| **S. No.** | **Variety/Line** | **11** | **11A** | **15-1** | **21** | **21A-2** | **24A** | **34-1** | **40A** | **40-1** | **40-2** | **40-3** | **42B** | **117** | **117A** | **117A-1** | **117-1** | **117-2** | **117-3** | **117-6** | **122** | **184** | **184-1** | **295** | **Postulated *Sr* genes** |
|  | CG1029 | R | R | R | R | R | R | R | R | R | R | R | R | R | R | R | R | R | R | MR | R | R | R | R | *Sr24+2+* |
|  | CG1036 | R | R | R | R | R | S | R | R | MR | R | R | R | R | R | MS | S | MS | MR | MR | R | MR | S | R | *Sr7b+* |
|  | DBW252 | R | R | R | R | R | R | R | R | R | R | S | R | R | R | R | R | R | R | R | R | R | R | R | *Sr8a+5+11+2+* |
|  | DDW47 (d)* | R | R | R | R | R | MS | R | R | R | MS | R | R | R | R | MR | S | R | NG | S | R | MS | R | R | *Sr11+7b+2+* |
|  | DDW48 (d) | R | R | R | R | R | S | R | R | R | R | R | MR | S | R | S | R | MS | S | MS | MR | R | R | S | *Sr7b+2+* |
|  | HD2733 | R | R | R | R | R | R | R | R | R | R | MR | R | R | R | R | R | R | R | R | R | R | R | R | *Sr31+2+* |
|  | HD2967 | R | R | R | R | R | R | R | R | S | R | MS | R | R | R | R | R | R | R | R | R | R | R | R | *Sr8a+11+2+* |
|  | HD3043 | R | R | R | R | R | R | R | R | R | R | R | R | R | R | R | R | R | R | R | R | R | R | R | *Sr31+2+* |
|  | HD3090 | R | R | R | R | R | R | R | R | R | R | R | R | R | R | R | R | R | R | R | R | R | R | R | *Sr31+2+* |
|  | HD3118 | R | R | R | R | R | R | R | R | R | R | S | R | R | R | R | R | R | R | R | R | R | R | R | *Sr9b+11+* |
|  | HD3171 | S | R | R | R | R | R | R | R | R | R | S | R | R | R | R | R | R | R | R | R | R | R | R | *Sr11+7b+2+* |
|  | HD3249 | R | R | R | R | R | R | R | R | R | R | MS | R | R | R | R | R | R | R | R | R | R | R | R | *Sr11+2+* |
|  | HD3293 | S | S | R | R | R | R | R | R | R | R | S | R | R | MR | R | R | R | R | R | R | R | R | R | *Sr13+2+* |
|  | HI1621 | R | R | R | R | R | R | R | R | R | S | MR | R | R | R | R | R | R | R | R | R | R | R | R | *Sr28+* |
|  | HI1628 | MR | R | R | R | R | R | R | R | R | R | MR | R | R | R | R | R | R | R | R | R | R | R | R | *Sr2+*R** |
|  | HI1633 | R | R | R | R | R | R | R | R | R | R | R | R | R | R | R | R | R | R | R | R | R | R | R | *Sr31+* |
|  | HI1634 | R | R | R | R | R | R | R | R | R | R | R | R | R | R | R | R | R | R | R | R | R | R | R | *Sr31+* |
|  | HI1650 | R | R | R | R | R | R | R | R | R | R | R | R | R | R | R | R | R | R | R | R | R | R | R | *Sr31+* |
|  | HI1653 | MR | R | R | R | R | R | R | R | R | MR | S | R | R | R | R | R | MR | R | MR | R | R | R | R | *Sr7b+* |
|  | HI1654 | MS | R | R | R | R | R | R | R | R | S | MS | R | R | MR | MR | R | R | R | MR | R | R | R | MR | *Sr13+* |
|  | HI1655 | R | R | R | R | R | R | R | R | R | R | R | R | R | R | R | R | R | R | R | R | R | R | R | *Sr2+*R |
|  | HI8498 (d) | MR | MR | R | R | R | S | R | R | R | R | R | S | S | R | S | S | S | S | S | MR | R | R | MR | *Sr11+2+* |
|  | HI8826 (d) | R | R | R | R | R | R | R | R | R | R | R | R | R | R | S | S | MR | R | S | R | MS | S | R | *Sr7b+2+* |
|  | HI8830 (d) | R | R | R | R | R | S | R | R | R | R | R | R | R | R | R | S | MS | R | MS | R | R | MS | R | *Sr7b+2+* |
|  | HPW349 | R | R | R | R | R | R | R | MR | R | MR | S | R | R | R | R | R | R | R | R | R | R | R | R | *Sr7b+2+* |
|  | HS507 | R | R | R | R | R | R | R | R | R | R | R | R | R | R | R | R | R | R | R | R | R | R | R | *Sr31+5+* |
|  | HS562 | R | R | R | R | R | R | R | S | MS | S | S | R | R | R | R | R | R | R | R | R | R | R | S | *Sr8a+9b+11+* |
|  | KRL19 | R | R | R | R | R | R | R | MR | R | R | R | R | R | R | R | R | R | R | R | R | R | R | R | *Sr8b+9b+11+2+* |
|  | KRL210 | S | S | R | R | R | R | R | MR | S | MS | S | MS | R | MS | R | R | S | MR | R | MR | R | R | S | *Sr7b+2+* |
|  | MACS4100 (d) | R | R | S | R | R | S | R | S | S | S | S | R | R | MS | S | S | S | S | S | R | S | S | R | *-* |
|  | MACS6768 | R | R | R | R | R | R | R | R | R | R | R | R | R | R | R | R | R | R | R | R | R | R | R | *Sr31+2+* |
|  | MP3288 | R | R | R | R | R | R | R | R | R | R | MS | R | R | R | R | R | R | R | R | R | R | R | R | *Sr24+2+* |
|  | MP3336 | MS | R | R | R | R | R | R | R | R | R | R | R | R | R | R | R | R | R | R | R | R | MR | S | *Sr11+2+* |
|  | NIAW3170 | R | R | R | R | R | R | R | R | R | MR | MR | R | R | R | R | R | R | R | R | R | R | R | R | *Sr8a+2+* |
|  | NIDW1149 (d) | R | MR | R | R | R | R | R | R | R | R | R | MR | R | R | R | R | R | R | R | R | R | MR | MR | *Sr11+2+* |
|  | PBW771 | R | R | R | R | R | R | R | R | R | R | R | R | R | R | R | R | R | R | R | R | R | R | R | *Sr31+2+* |
|  | RAJ4083 | R | R | R | R | R | R | R | R | MR | R | S | R | R | R | R | R | R | R | R | R | R | R | R | *Sr11+* |
|  | VL2041 | S | R | R | R | R | R | R | R | R | R | S | R | R | R | R | R | R | R | R | R | R | R | R | *Sr30+5+11+* |
|  | VL907 | R | R | NG | R | R | R | R | R | R | R | R | R | R | R | R | R | R | R | R | R | R | R | R | *Sr31+2+* |
|  | WH1124 | R | R | R | R | R | R | R | R | R | R | R | R | R | R | R | R | R | R | R | R | R | R | R | *Sr7b+2+* |
| *** (d): durum wheat; ** resistant to all pathotypes | | | | | | | | | | | | | | | | | | | | | | | | | |

**Supplementary Figure 1. Amplification profiles of forty wheat varieties against *Sr2* linked SSR marker Xgwm533 on agarose gel. M: 100 bp ladder (HiMedia Laboratory Pvt. Ltd., India); 1-40: wheat varieties arranged as per the S. No. in Table 1; -ve: negative controls for *Sr2*; -+ve: positive control for *Sr2*. The 120 bp fragments indicate presence of *Sr2*.**
